# Direct Ionic Regulation of the Activity of *Myo*-Inositol Biosynthesis Enzymes in Mozambique Tilapia

**DOI:** 10.1371/journal.pone.0123212

**Published:** 2015-06-11

**Authors:** Fernando D. Villarreal, Dietmar Kültz

**Affiliations:** EcoPhysiological Proteomics Laboratory, Department of Animal Science, University of California Davis, One Shields Avenue, Davis, California 95616, United States of America; Institut National de la Recherche Agronomique (INRA), FRANCE

## Abstract

*Myo*-inositol (Ins) is a major compatible osmolyte in many cells, including those of Mozambique tilapia (*Oreochromis mossambicus*). Ins biosynthesis is highly up-regulated in tilapia and other euryhaline fish exposed to hyperosmotic stress. In this study, enzymatic regulation of two enzymes of Ins biosynthesis, Ins phosphate synthase (MIPS) and inositol monophosphatase (IMPase), by direct ionic effects is analyzed. Specific MIPS and IMPase isoforms from Mozambique tilapia (MIPS-160 and IMPase 1) were selected based on experimental, phylogenetic, and structural evidence supporting their role for Ins biosynthesis during hyperosmotic stress. Recombinant tilapia IMPase 1 and MIPS-160 activity was assayed *in vitro* at ionic conditions that mimic changes in the intracellular milieu during hyperosmotic stress. The *in vitro* activities of MIPS-160 and IMPase 1 are highest at alkaline pH of 8.8. IMPase 1 catalytic efficiency is strongly increased during hyperosmolality (particularly for the substrate D-Ins-3-phosphate, Ins-3*P*), mainly as a result of [Na^+^] elevation. Furthermore, the substrate-specificity of IMPase 1 towards D-Ins-1-phosphate (Ins-1*P*) is lower than towards Ins-3*P*. Because MIPS catalysis results in Ins-3*P* this results represents additional evidence for IMPase 1 being the isoform that mediates Ins biosynthesis in tilapia. Our data collectively demonstrate that the Ins biosynthesis enzymes are activated under ionic conditions that cells are exposed to during hypertonicity, resulting in Ins accumulation, which, in turn, results in restoration of intracellular ion homeostasis. We propose that the unique and direct ionic regulation of the activities of Ins biosynthesis enzymes represents an efficient biochemical feedback loop for regulation of intracellular physiological ion homeostasis during hyperosmotic stress.

## Introduction

With very few exceptions (some halophilic archaea), all cells maintain intracellular inorganic ion homeostasis within narrow limits and studies directed at the mechanisms by which such homeostasis is maintained during extracellular osmotic stress are of ubiquitous interest [1, 2]. Plant and bacterial cells subjected to droughts or altered soil composition, renal inner medullary cells of mammals, and epithelial cells of aquatic organisms that inhabit variable salinity environments (estuaries, desert lakes) are all equipped with a high physiological capacity for maintaining intracellular inorganic ion homeostasis [3–7]. In animals, a high physiological capacity for responding to hypertonic stress depends on the ability for compensating passive loss of water across the semi-permeable cell membrane by 1) regulatory volume increase to restore cell volume homeostasis followed by 2) replacement of excessive intracellular inorganic ions by compatible organic osmolytes to restore intracellular electrolyte homeostasis [3, 6, 8, 9]. To avoid and alleviate macromolecular crowding during hypertonic stress, cell volume is rapidly restored when disturbed by hypertonic stress (within seconds to minutes). This restoration of cell volume is a result of activation of inorganic ion uptake, which is mediated largely by sodium-coupled secondarily active transporters, including Na^+^/K^+^/2Cl^-^ (NKCC) cotransporters, and Na^+^/H^+^ exchangers (NHE) [10, 11]. Although restoring cell volume by creating an osmotic gradient for water to follow passively into cells, this active uptake of inorganic ions increases intracellular ionic strength, which is detrimental for cell function, e.g. by interfering with normal protein folding and activity [12]. In contrast to inorganic electrolytes, organic osmolytes (sugars and other polyols, methylamines, amino acids) are compatible with normal cell function over a wide concentration range [2, 9, 13]. The intracellular concentration of compatible organic osmolytes is adaptively regulated by adjustment of their synthesis, degradation, or transport across the plasma membrane [14–17]. In particular, transport of extracellular Ins is mediated through sodium/Ins (SMIT) [18] and hydrogen/Ins (HMIT) [19] cotransporters.

*Myo*-inositol (Ins) belongs to the group of compatible organic osmolytes referred to as cyclic polyols, which are represented in all domains of life [2, 13]. Ins biosynthesis involves two enzymes: (1) D(L)-*myo*-inositol-3(1)-phosphate synthase (MIPS, EC 5.5.1.4) catalyzes the conversion of glucose 6-phosphate to *myo*-D(L)-inositol-3(1)-phosphate [20], and (2) inositol monophosphatase (IMPase, EC 3.1.3.25), which dephosphorylates inositol phosphate to yield Ins [21]. Both enzymes have been thoroughly characterized in a number of organisms and several high-resolution 3D protein structures from multiple species have been experimentally determined for these proteins (S1 Table in Supporting Information). Conserved features of the protein structure for MIPS include a Rossman fold (NAD^+^ binding motif), a tetramerization/catalytic domain, and a central domain, with an overall homotetrameric quarternary arrangement [20]. *Saccharomyces cerevisiae* MIPS requires NAD^+^ for catalysis, although no net production of NADH is observed, since NADH represents an intermediate, which is recycled back to NAD^+^ during each catalytic cycle [22]. In mammals, at least three splice variants of MIPS have been identified that show a high degree of sequence and structural conservation to MIPS from lower organisms [23]. Enzymatic activity of MIPS homologous from all species tested is potently and specifically inhibited by micromolar concentrations of substrate analogues such as 2-deoxy-glucose 6-phosphate (2dG6P) and 2-deoxy glucitol 6-phosphate [20].

IMPase high-resolution 3D structures have also been experimentally solved for many species, including human and bovine [21]. In contrast to MIPS, IMPase is typically arranged as a homodimer, with each monomer comprised of a five-layer αβαβα sandwich. To be catalytically active IMPase requires a divalent cation (such as Mg^2+^) as a co-factor. Many species have multiple genes encoding distinct IMPase isoforms and the substrate specificity of IMPase isoforms is somewhat flexible in that these enzymes can dephosphorylate several inositol monophosphate isomers (Ins 1-, 3-, 4- and 6-P) [24]. Li^+^ is a known inhibitor of IMPase, with an IC_50_ ranging from 0.7 to 30 mM (BRENDA database, [25]). Additionally, biphosphonates such as the L690,330 compound are potent inhibitors of IMPase enzymes at micromolar concentrations [26].

Recently, we have identified two MIPS splice variants for tilapia (MIPS-160 and MIPS-250) that are encoded at a single genomic locus [27]. Moreover, MIPS-160 and IMPase 1 are highly up-regulated at mRNA and protein levels in response to elevated environmental salinity in multiple tissues of Mozambique tilapia, Nile tilapia (*O*. *niloticus*) and eel (*Anguilla anguilla*) [27–30]. Increased enzymatic IMPA activity and Ins accumulation in response to elevated salinity have also been observed in multiple tilapia tissues *in vivo* [28, 29, 31]. These observations provide evidence for Ins being a physiologically important organic osmolyte that protects euryhaline fish during salinity stress. However, the time course for increasing the abundance of MIPS and IMPA is slow (hours to days) relative to the need for starting to accumulate organic osmolytes within minutes of hypertonicity (see above). Therefore, in this work we have designed and conducted experiments to test the hypothesis that MIPS and IMPA enzymatic activity can be directly increased by alteration of inorganic ion concentrations that reflect the conditions experienced by cells exposed to hypertonicity.

## Materials and Methods

### Phylogenetic analyses of MIPS and IMPA sequences and structures

MIPS and IMPase sequences from different species (accession numbers in S3 and S4 Tables in Supporting Information) were retrieved from NCBI databases for most species (www.ncbi.nlm.nih.gov), and from ENSEMBL databases for three spined stickleback (*Gasterosteus aculeatus*), using BLAST and BLAST/BLAT tools, respectively. Bidirectional best hit [32] was performed using BLAST (or BLAST/BLAT for *G*. *aculeatus*), with both human (NM_005536.3) and *O*. *niloticus* (XP_003439317.1) sequences as anchors, to determine putative IMPase orthologues in other species (S2 Table in Supporting Information). For multiple sequence alignment (MSA), T-COFFEE server (tcoffee.vital-it.ch) was used [33]. Maximum parsimony phylogenetic trees were built using Phylip PROTPARS with sequence input randomization (10 jumbles) and bootstrapping procedure (500 replicates) for branch support, through POWER server [34]. Using MIPS-160 and IMPase 1 sequences as queries, the structural 3D models were constructed using the I-TASSER server [35] with default options. Calculated 3D structure models were superimposed to experimentally determined models of known homologs using Swiss-Pdb viewer 4.0.4 software (http://spdbv.vital-it.ch/) and figures were rendered using Jmol (www.jmol.org). ConSurf server was used to graphically overlay the conservation of amino acids at every position, based on their phylogenetic relationships, over 3D protein structure models [36]. For this purpose, MIPS-160 and IMPase 1 calculated 3D models were used as queries in combination with the corresponding MSA and phylogenetic trees (S1 and S2 Figs in Supporting Information). For analysis of primary sequence features the ProtParam tool (web.expasy.org/protparam) was used [37].

### MIPS-160 and IMPase 1 cloning and protein expression

Total RNA from seawater acclimated *O*. *mossambicus* gills [27] was extracted using Trizol reagent (Invitrogen Life Technologies, Carlsbad, CA) following vendor instructions. cDNA was generated using random hexamer primers (Promega, Madison, WI) and Superscript III reverse transcriptase (Invitrogen Life Technologies). Primers for specifically amplifying the full-length coding sequences of MIPS-160 and IMPase 1 (sequences in S5 Table in Supporting Information) were designed based on Genbank entries DQ465381.1 (MIPS-160) and AY737046.1 (IMPase 1) [30, 38]. Forward and reverse primers were designed to add Nhe I and Xho I restriction sites to *O*. *mossambicus* MIPS-160 and IMPase 1 cDNAs to enable ultimately cloning those cDNAs in frame with an amino-terminal hexa-His tag into the pET24a vector (EMD Biosciences, San Diego, CA). As an intermediate step, PCR products were first cloned into TOPO vector by TA cloning (Invitrogen Life Technologies) and plasmids amplified in *E*. *coli* One Shot TOP10 cells (Invitrogen Life Technologies), grown on LB agar plates supplemented with 100 μg·mL^-1^ Ampicillin (Sigma, St Louis, MO). Plasmids (MIPS-160-TOPO and IMPase 1-TOPO) were isolated using QIAprep Spin Miniprep Kit (Qiagen, Valencia, CA) and sequenced by the UC Davis Sequencing facility with an ABI Prism 3730 Genetic Analyzer. Plasmids containing the validated sequences were then subjected to Nhe I-Xho I restriction (New England Biolabs, Beverly, MA). The inserts were purified from gels using a QIAquick Gel Extraction Kit (Qiagen, Valencia, CA), and purified inserts were directionally subcloned into pET24a (previously digested with Nhe I-Xho I) using T4 ligase (Promega). Ligated pET24a constructs were transformed into *E*. *coli* TOP10 cells and plated on LB plates supplemented with 50 μg·mL^-1^ Kanamycin (Sigma). Single colonies were picked and correct insert sequences confirmed by PCR and restriction enzyme digestion. Validated MIPS-160-pET24a and IMPase 1-pET24a plasmids were transformed into the *E*. *coli* expression strain Rosetta 2(DE3)pLys (EMD Biosciences) and plated on LB plates containing chloramphenicol (34 μg·mL^-1^, Sigma) and kanamycin. Three mL of LB (supplemented with chloramphenicol-kanamycin) were inoculated with single colonies of Rosetta 2(DE3)pLysS bearing IMPase 1-pET24a or MIPS-160-pET24a, and propagated for 16 h at 37°C in an orbital shaker (200 x rpm). Overnight cultures were used to inoculate larger LB cultures (supplemented with the same antibiotics) at a ratio of 1:100. Bacterial cultures were then incubated at 37°C until an OD_600_ of 0.6–1 was reached. The cultures were then induced by addition of IPTG (Promega, 1 mM final concentration) and incubated for another 16 h at 37°C in an orbital shaker (200 x rpm). IPTG-induced cultures were collected in centrifuge tubes, kept on ice for 15 min, and centrifuged for 5 min at 5,000 x rpm. The supernatant was discarded and bacterial pellets frozen at -80°C for further use. For both IMPase 1 and MIPS-160, the proteins were not forming inclusion bodies (data not shown), and were consequently purified from the soluble fraction.

### Recombinant MIPS-160 purification

Induced bacterial pellets were resuspended by adding 1 mL of BugBuster reagent (EMD Biosciences) per 20 mL of culture from which the pellet was derived. To remove nucleic acids, 1 μL of Benzonase (EMD Bioscience) was added per mL of BugBuster. After shaking for 20 min at room temperature, 0.25 volumes of 5X MIPS binding buffer (1X final concentration = 50 mM Tris-HCl, pH 8.2, 50 mM NaCl, 30 mM imidazole) were added to the lysate. Protease inhibitor (Complete Mini Tablet, Roche, Indianapolis, IN) was also added (one mini-tablet per 15 mL of buffer). For immobilized metal-affinity chromatography (IMAC), the equilibrated lysate was injected into one 1 mL-HisTrap FF Ni^2+^ column (GE Healthcare), previously washed and equilibrated with 10 column volumes (CV) of 1X MIPS binding buffer, by using a peristaltic pump (flow rate = 1 mL min^-1^). Afterwards, 45 mL of wash buffer (WB, 50 mM Tris-HCl, pH 8.2, 50 mM NaCl, 30 mM imidazole, 0.5% Tween-20) were injected into the column. Protein recovery was performed with 5 mL of elution buffer (50 mM Tris HCl pH 8.2, 50 mM NaCl, 300 mM imidazole), supplemented with the inhibitor protease cocktail. The eluate was diluted 3 times in 10 mM Tris-HCl, pH 8.2, for further purification by ion exchange chromatography (IEX). A 1 mL HiTrap QFF column (GE Healthcare) was equilibrated with 5 CV of IEX Start buffer (10 mM Tris-HCl, pH 8.2, 50 mM NaCl), followed by 5 CV of 1M Elution buffer (10 mM Tris-HCl, pH 8.2, 1 M NaCl), and washed with 5 CV of IEX Start Buffer. The diluted eluate from the preceding HisTrap purification was injected into the treated QFF column, which was then washed with 15 CV of IEX Wash buffer (10 mM Tris-HCl, pH 8.2, 150 mM NaCl). Finally, recombinant MIPS-160 was eluted with 5 CV of IEX Elution buffer (10 mM Tris-HCl, pH 8.2, 220 mM NaCl). The resulting eluate was diluted with 1 volume of Storage buffer (50 mM Tris-HCl, pH 8.2, 2 mM NH_4_Cl, 0.2 mM DTT). Buffer exchange and protein concentration was performed with Amicon ultrafiltration device (MWCO: 3,500 Da, EMD Millipore, Billerica, MA) using 5 centrifugation cycles of 30 min at 4°C and 5,000g. At the end of each cycle, new storage buffer was added to facilitate buffer exchange. Finally, the concentrated protein (concentration factor: ~20x) was aliquoted and stored at -80°C until use.

### Recombinant IMPase 1 purification

Bacterial pellets were lysed in BugBuster supplemented with Benzonase as described above for MIPS-160. Subsequently, 0.25 volumes of 5X IMPA binding buffer (1X final concentration: 50 mM Tris-HCl, pH 7.4, 50 mM NaCl, 60 mM imidazole) were added, supplemented with one mini-tablet EDTA free protease inhibitor cocktail (Roche) per 15 mL of buffer. The lysate was injected into a 1 mL HisTrap column, previously equilibrated with 10 CV of 1X IMPA binding buffer. The column was successively washed with 10 CV of three wash buffers (50 mM Tris-HCl, pH 7.4, 50 mM NaCl), with increasing imidazole concentration (Wash 1: 60 mM, Wash 2: 75 mM, Wash 3: 90 mM). Finally, IMPase 1 was eluted with Elution buffer (50 mM Tris-HCl, pH 7.4, 50 mM NaCl, 500 mM imidazole). Eluate was diluted with 1 volume of storage buffer (50 mM Tris-HCl, pH 7.4, 50 mM NaCl, 1 mM MgCl_2_) and concentrated as described for MIPS-160. Protein concentration was determined by bicinchoninic acid assay (Pierce Thermo Scientific, Rockford, IL).

### SDS-PAGE and Western blotting

Protein samples were combined with 6X Laemmli's Loading dye [39] and heated at 90°C for 5 min before loading on 15%-acrylamide gels. Gels were visualized by colloidal Coomassie Blue staining for total protein visualization as previously described [40], or used for blotting onto PVDF membranes (BioRad, Hercules, CA) for identification by antibodies. A Semi-Dry trans-blot apparatus (BioRad) was used for immunoblotting. PVDF membranes were blocked with 10% non-fat dry milk in Tris Buffer saline + 0.5% Tween-20 (TBS-T) overnight at 4°C. After three washes with 0.5% milk in TBS-T (5 min each), membranes were exposed for 1 h at RT to primary antibodies. Mouse monoclonal Anti-His (Pierce Thermo Scientific) diluted 1:3000, rabbit polyclonal Anti-Human Impa1 (Santa Cruz Biotechnology, Santa Cruz, CA) diluted 1:500, or goat polyclonal anti-Human Isyna1 (Santa Cruz Biotechnology) diluted 1:250 in TBS-T plus 3% bovine serum albumin (BSA) were used. Antibody solution was removed and membrane was washed three times with 0.1% BSA in TBS-T. Membranes were then exposed to secondary antibodies (diluted in 3% BSA TBS-T) 1:15000 fold (anti-mouse-HRP, Pierce Thermo Scientific), or 1:5000 fold (goat anti-rabbit-HRP and donkey anti-goat-HRP, Santa Cruz Biotechnology), respectively, for 1 h at RT. After three 5 min washes with TBS-T, membranes were developed with West Pico (anti-His) or West Femto (anti-Impa1 and anti-Isyna1) chemiluminiscence reagents (Pierce Thermo Scientific).

### Enzyme activity assays

MIPS-160 activity was assayed as described previously [41], with modifications noted below. If not otherwise stated, the basic enzyme reaction took place in MIPS assay buffer (MAB, final concentration = 50 mM Tris-HCl, pH 8.2, 2 mM NH_4_Cl and 0.2 mM DTT). MAB was combined with different concentrations of G6P (Sigma) and the reaction was initiated by addition of the purified MIPS-160 enzyme (final concentration 0.061 μg μL^-1^, 1 μM). Reactions were incubated at 28°C, and 25 μL aliquots were taken at selected time points, and combined with 10 μL of 20% TCA to stop the reaction. The reaction aliquots were then incubated for 10 minutes on ice, and centrifuged at 14,000 x rpm for 10 min. The supernatant (35 μL) was transferred to a new tube, followed by addition of 40 μL of 0.2 M NaIO_4_, brief vortexing, and incubation at 37°C for 2 h. To neutralize any unreacted NaIO_4_, 40 μL of 1M Na_2_SO_3_ were added. Samples were vortexed briefly and used for determination of free inorganic phosphate (Pi) (see below).

IMPase 1 activity was determined in IMPA assay buffer (IAB) consisting of 50 mM Tris-HCl, pH 8.2, 50 mM NaCl and 1 mM MgCl_2_, unless otherwise stated (modified from [42]). 2X IAB buffer was combined with substrate, eitherD-Ins-1-phosphate, Ins-1*P*, or D-Ins-3-phosphate, Ins-3*P* (Cayman Chemicals, Ann Arbor, MI) at different concentrations, and the reaction was started by addition of the purified enzyme (final concentration 0.0036 μg μL^-1^, 114 nM). Reactions were incubated at 28°C, and aliquots (25 μL) were taken at selected time points, and transferred to tubes containing 10 μL of 10% TCA to stop the reaction. Aliquots were incubated 10 min on ice, and centrifuged as described for the MIPS-160 activity assay. After centrifugation, supernantant was transferred to a new tube containing 80 μL of MilliQ water, followed by briefly vortexing before determining Pi content.

Inorganic phosphate (Pi) content was determined by using BioMol Green reagent (Enzo Life Sciences, Farmingdale, NY). Samples were transferred to wells in 96-microplates (50 μL) and 150 μL of BioMol Green was added to each well. Microplates were incubated for 10 min at room temperature in a microplate shaker (400 x rpm), and absorbance at 620 nm recorded in a microplate reader (TECAN, Crailsheim, Germany). A standard curve was generated using known KHPO_4_ concentrations.

For assaying enzyme activity at different ionic strengths, buffers were adjusted by adding corresponding amounts of NaCl or KCl stock solutions. MAB buffer osmolality was 124 ± 2.6 mOsmol/kg and IAB buffer osmolality was 220 ± 2.8 mOsmol/kg. Osmolality was measured using a Vapor Pressure Osmometer (Wescor Vapro 5520, Wescor Inc., Logan, UT).

To determine kinetic parameters of MIPS-160 and IMPase 1, initial velocities V_o_ were calculated for each substrate concentration at every condition tested. V_o_ values were fitted to a Michaelis-Menten expression by non-linear least-squares-regression analysis using GraphPad 5 software (GraphPad, San Diego, CA), and used to determine *K*_M_, V_max_ and *k*_*cat*_.

## Results

### Characterization of IMPase 1 and MIPS-160 by bioinformatic tools

The overall objective of this study was to achieve *in vitro* reconstitution of enzymatic activity of two key enzymes in the *myo*-inositol biosynthesis pathway of tilapia to determine direct ionic and osmotic effects on enzyme function. The corresponding cDNAs were selected based on experimental evidence for their strong hyperosmotic induction in several tilapia tissues [27–29]. However, before cloning these cDNAs and investing significant resources into their recombinant expression and purification, their phylogenetic relationship to vertebrate homologues and the structure of the IMPase 1 and MIPS-160 proteins were analyzed. This was necessary because at least four *IMPase* loci are present in the fully sequenced Nile tilapia genome [29], and two MIPS variants have been observed previously in tilapia [27, 28].

**Fig 1:**
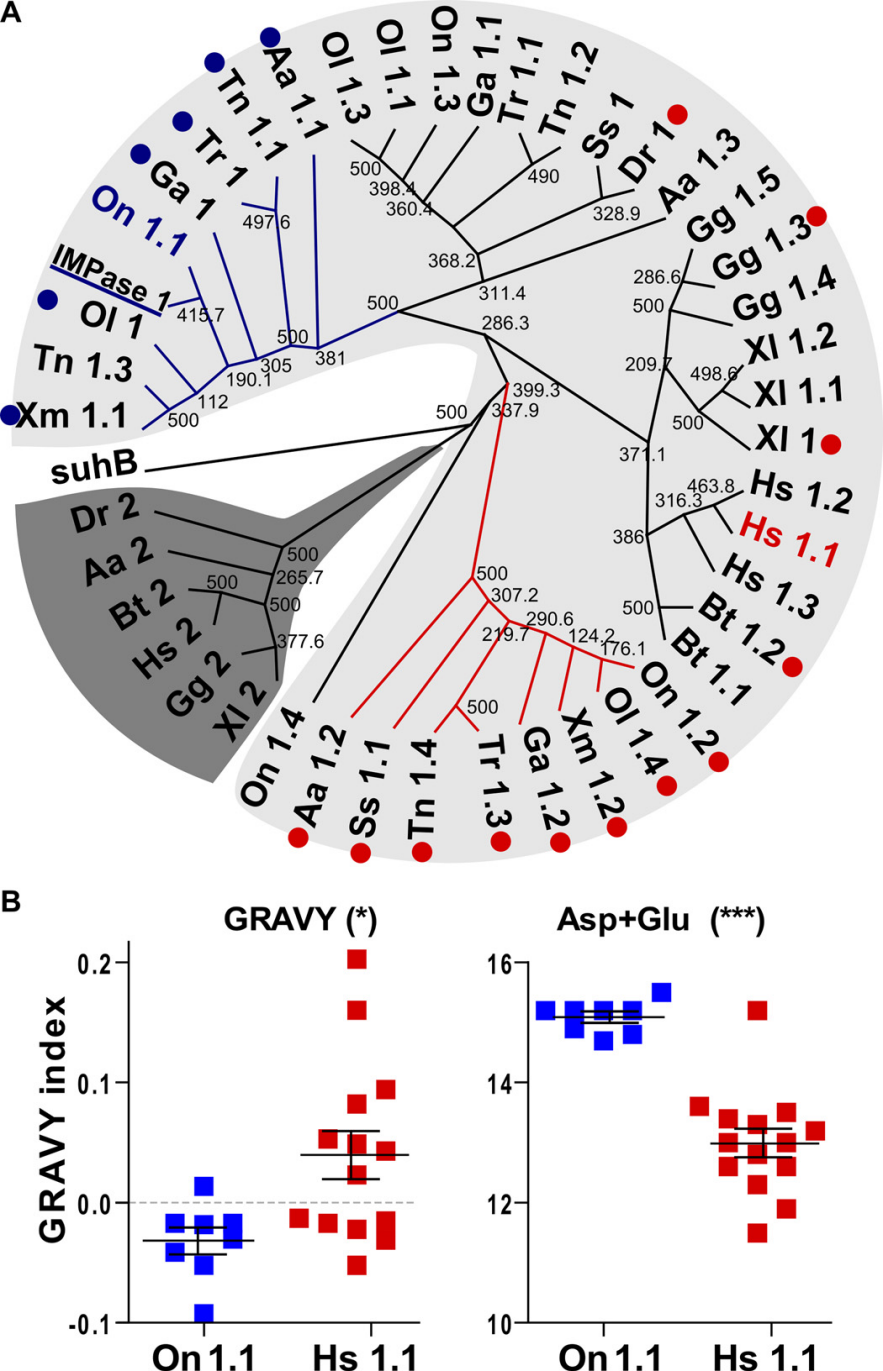
Phylogenetic tree of vertebrate IMPase proteins. (**A**) Full-length protein sequences of IMPase isoforms from *Homo sapiens* (Hs), *Bos taurus* (Bt), *Gallus gallus* (Gg), *Xenopus laevis* (Xl) and fish: *Anguilla anguilla* (Aa), *Danio rerio* (Dr), *Gasterosteus aculeatus* (Ga), *Oryzias latipes* (Ol), *O*. *mossambicus* (Om), *O*. *niloticus* (On), *Salmo salar* (Ss), *Tetraodon nigroviridis* (Tn), *Takifugu rubripes* (Tr) and *Xiphophorus maculatus* (Xm) were retrieved from the corresponding genomes. Results for best bi-directional BLAST are shown in S2 Table in Supporting Information, and accession numbers used to build the tree are available in S3 Table in Supporting Information. The tree was built by maximum parsimony (bootstrap values for 500 replicates are shown). In light gray, the IMPase 1 clade is highlighted, while the dark gray clade corresponds to the IMPase 2 clade. Red dots denote that a sequence is orthologous to the human *Hs* 1.1 (by bidirectional best BLAST hit), while blue dots depict fish sequences that are orthologues to the Nile tilapia On 1.1 sequence.. The latter sequences fall in a separate subclade (blue branches). Labelling of the sequences correspond to the species followed by the numbers assigned in their corresponding annotation in databases, except for *O*. *mossambicus* IMPase 1 (underlined). (**B**) Analysis of primary sequence of orthologues to Hs 1.1 (red) and to On 1.1 (blue). Grand average of hydrophobicity (GRAVY) and overall contents of negatively charged amino acids (Glu and Asp) in each sequence are shown. Asterisks represent significant differences analyzed by two tailed t-test (***, *P*<0.0001; * *P*<0.05).

In fish, several IMPase isoforms (annotated as IMPase 1) can be retrieved after searches in databases (S3 Table in Supporting Information). A phylogenetic tree using sequences from several species (Fig 1A) shows that the osmoregulated Mozambique tilapia isoform (Om IMPase 1) belongs to an IMPase 1 clade, in particular to a sub-clade (blue branches) containing the putative immediate orthologues of On 1.1 (blue dots, S2 and S3 Tables in Supporting Information). This sub-clade contains the hyperosmotic-stress responsive isoforms from *Oreochromis niloticus* (IMPase On 1.1), *Anguilla anguilla* (Aa 1.1) [29] and *Gasterosteus aculeatus* (Ga 1). IMPase 1.1 protein abundance in gills of three-spine sticklebacks also increases significantly during high salinity stress (unpublished data). A second fish IMPase 1 sub-clade (red branches) containing putative orthologues of human IMPase 1.1 (red dots), includes members from euryhaline and stenohaline fish species, except for the sequence from *D*. *rerio*. This *D*. *rerio* sequence groups in another clade containing other fish sequences, which are not orthologous to neither the human IMPase 1.1 nor the *O*. *niloticus* 1.1 sequences. With the exception of eel and zebrafish, none of the fish species analyzed here contained members of the IMPase 2 clade (Fig 1A). The comparative analysis of the primary sequences that are orthologues to Hs 1.1 (red dots) and On 1.1 (blue dots), respectively, revealed that the On 1.1 subclade is characterized by a reduced content of hydrophobic amino acids and an increased number of negatively charged amino acids (Fig 1B).

**Fig 2:**
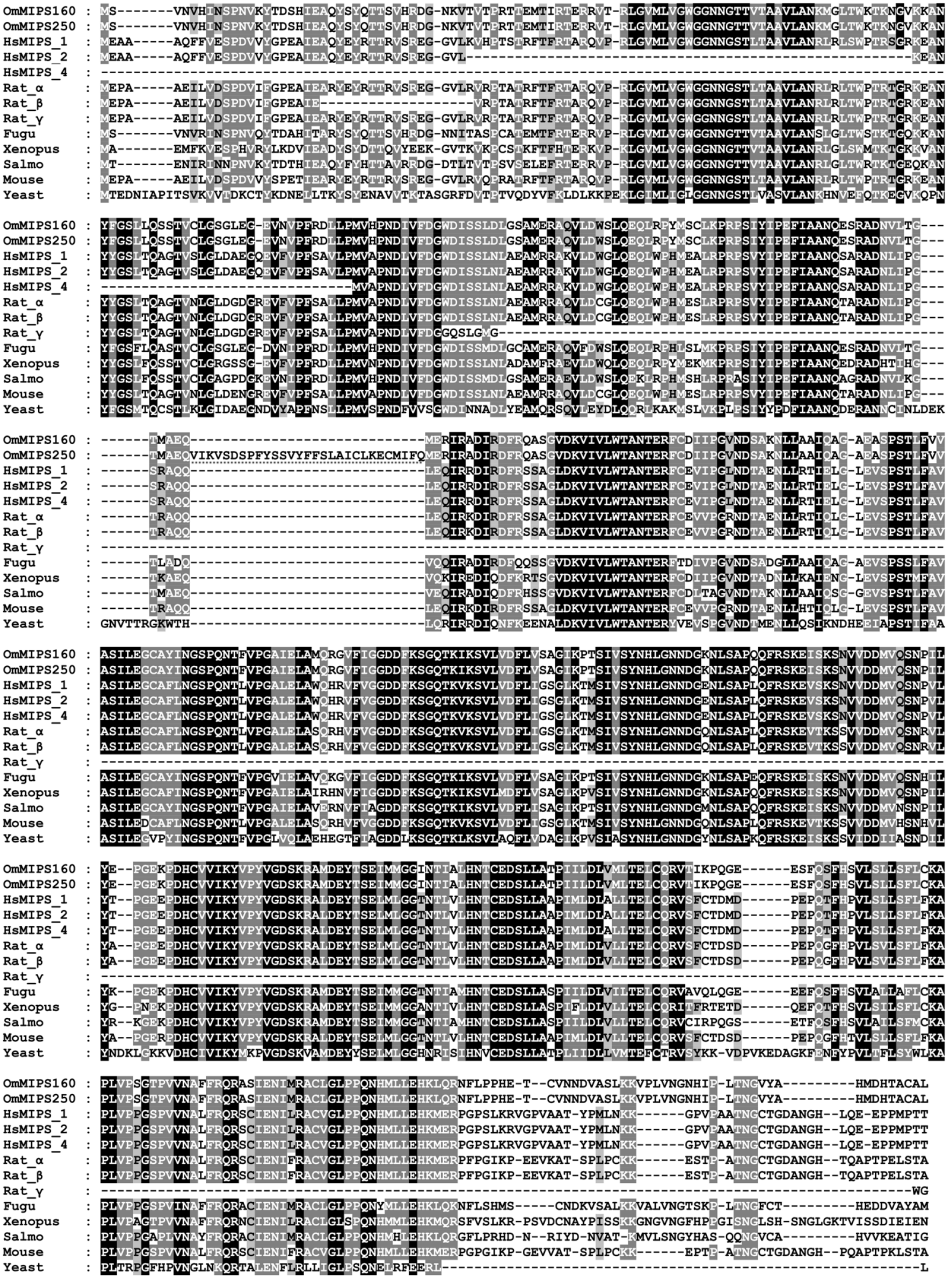
Multiple sequence analysis of MIPS isoforms from different species. MIPS sequences from different species (including three splicing variants from human, Hs, three from rat, and two known alternative variants from Mozambique tilapia, MIPS-160) were aligned using T-Coffee. A unique portion of the MIPS-250 variant that is not shared by any other sequence is highlighted (in block 3 from top). Accession numbers used to build the MSA are available in S4 Table in Supporting Information.

In contrast to IMPase, only one *MIPS* genomic locus is present in fish. However, several transcript variants have been observed in human and rat [23, 43]. In Mozambique tilapia, two variants have been reported that are likely the result of alternative splicing during mRNA maturation [27, 28]. The multiple sequence alignment depicted in Fig 2 illustrates that the long variant (MIPS-250) has a region not shared with any other of the sequences included in the analysis (this region is encoded by a retained intron). Because the short MIPS-160 variant shares a higher degree of overall sequence conservation with MIPS from other species it was chosen over the MIPS-250 longer variant for recombinant expression. Additionally, the short MIPS-160 variant has previously been shown to increase more strongly during hyperosmotic stress in both gills and brain of tilapia [27, 28].

**Fig 3:**
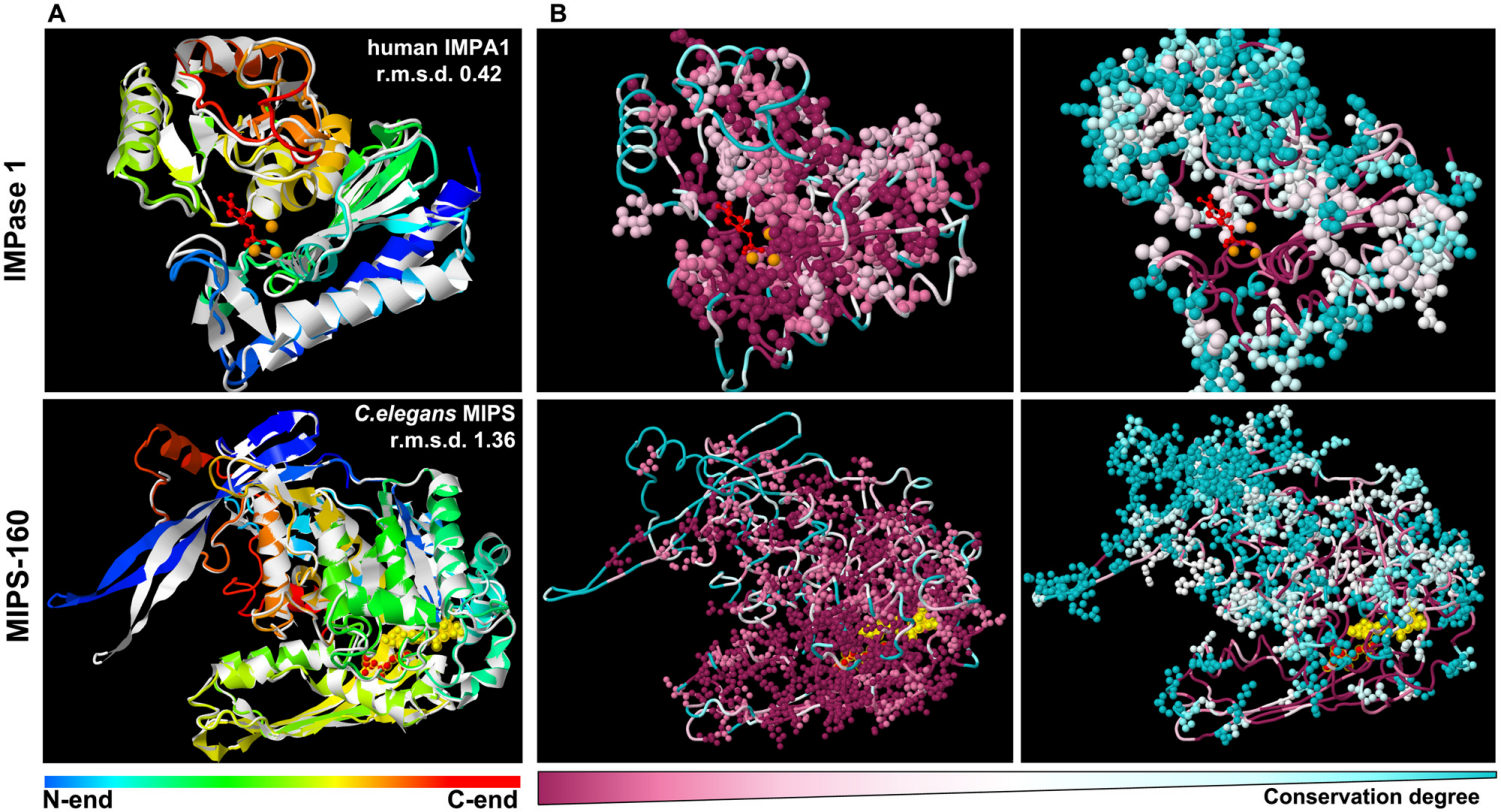
IMPase 1 and MIPS-160 3D structural models and conservation. (**A**) 3D models for both IMPase 1 (top) and MIPS-160 (bottom) were generated using the I-TASSER server. Models (colored from N- to C-end) are shown superimposed to the 3D structure with the best r.m.s.d value for each model (in white). (**B**) Conservation of amino acids at the structural level (generated by CONSURF). Using the IMPase 1 and MIPS-160 models, the most highly conserved amino acid residues (levels 9 to 7) are shown in the left panel, while the less conserved amino acids (levels 6 to 1) are shown in the right. Additionally, in (**A**) and (**B**), co-crystallized catalysis-relevant molecules are shown: *IMPase 1*, Ins monophosphate (red) and Mg^2+^ ions (orange); MIPS-160: NAD^+^ (yellow), 2-D-glucitol phosphate (red).

To evaluate the conservation of IMPase 1 and MIPS-160 three-dimensional structures, 3D structural models were generated based on experimentally solved structures of homologues from other species. The resulting structures (Fig 3A) are shown superimposed to the best corresponding reference structure: human IMPase 1, PDB 2HHM (r.m.s.d. 0.42) and *C*. *elegans* MIPS 1VKO (r.m.s.d. 1.36), confirming that the primary sequence similarity is reflected in the three-dimensional structure (S1 Table in Supporting Information). Analysis of the modelled tilapia IMPase 1 and MIPS-160 structures with the CONSURF tool reveals that the conservation of amino acids surrounding the active site is higher than that of amino acids exposed to the solvent (Fig 3B, S1 and S2 Figs in Supporting Information. These bioinformatic data, in conjunction with previous experimental evidence, were used as a basis for selecting IMPase 1 and MIPS-160 proteins to analyze direct ionic and osmotic effects on their activity.

### Purified MIPS-160 and IMPase 1 show enzymatic activity

**Fig 4:**
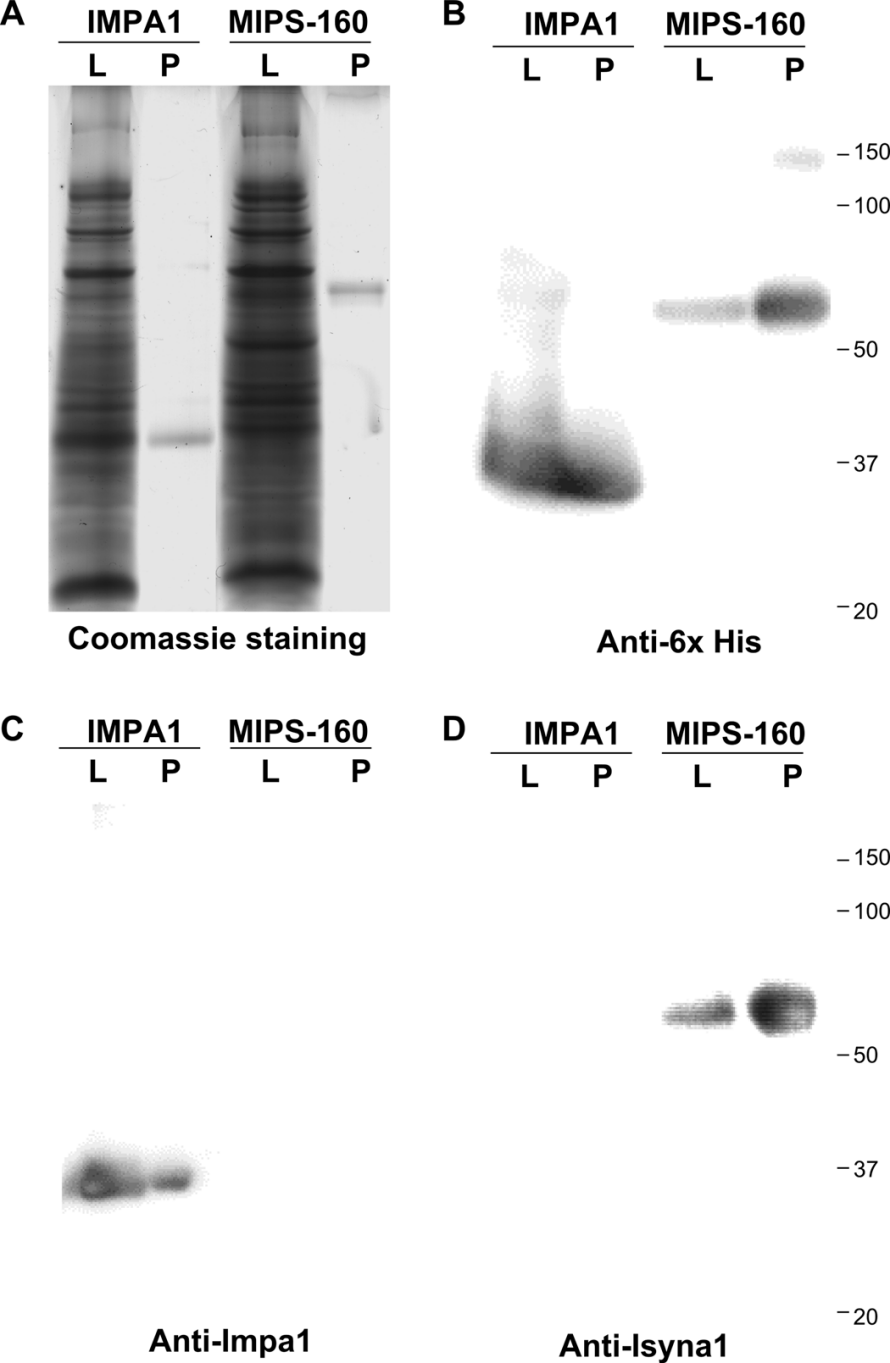
Recombinant purified proteins are recognized by specific antibodies. The proteins were detected in both the bacterial lysates following induction (L) and after the purification process (P) (**A**). Following protein induction and purification, recombinant MIPS-160 and IMPase 1 were specifically recognized by the Anti-6x His tag antibody (**B**), while each protein was selectively recognized by antibodies raised against the human orthologues: Impa1 (**C**) or Isyna1 (**D**).

MIPS-160 and IMPase 1, cloned from Mozambique tilapia gill cDNA and fused to a carboxy-terminal 6x-His tag, were expressed in *E*. *coli*, and purified using IMAC for IMPase 1, or IMAC followed by IEX for MIPS-160 (Fig 4A). Purified proteins had the expected molecular mass (IMPase 1, 32 kDa; MIPS-160, 61 kDa), and their identity was confirmed by Western blot using anti-6x His tag monoclonal antibody (Fig 4B). Moreover, both proteins are recognized by specific polyclonal antibodies raised against their human homologues (Fig 4C and 4D).

**Fig 5:**
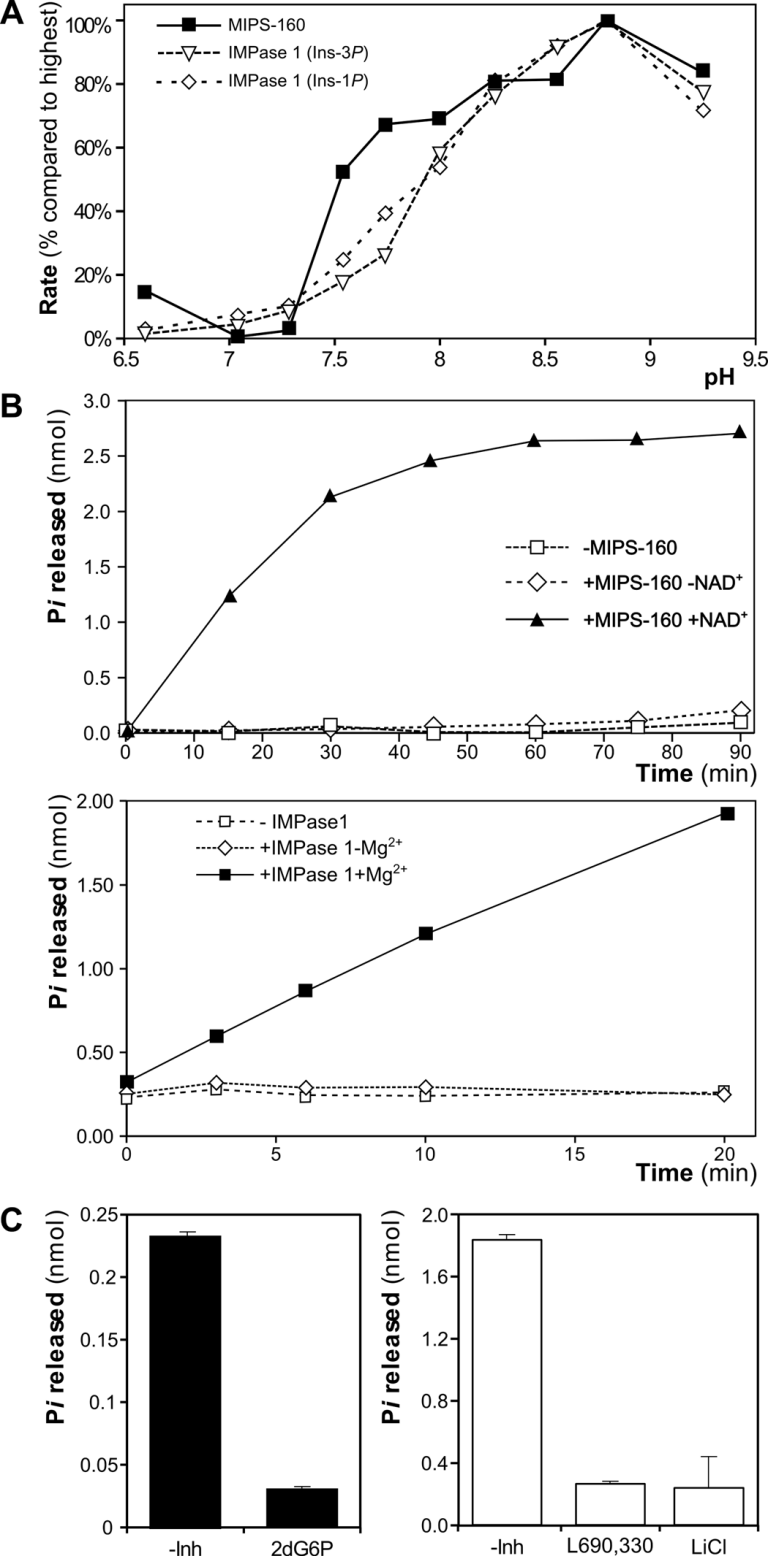
Enzymatic activity of purified IMPase 1 and MIPS-160. (**A**) MIPS-160 and IMPase 1 were incubated with their respective substrates at different pH, and reaction rate was measured for each point. Values are expressed relative to the highest activity observed (for both enzymes, at pH 8.8). (**B**) Enzymatic activity of both MIPS-160 (top) and IMPase 1 were determined with or without their known required cofactors (NAD^+^ and Mg^2+^, respectively). (**C**) Known MIPS and IMPase inhibitors (500 μM) significantly reduce the activity of MIPS-160 (100 μM 2-deoxy-G6-*P* [2dG6P]) and IMPase 1 (Ins-1*P* as substrate, 50 μM L690,330 or 5 mM LiCl as inhibitors).

The activity of the purified recombinant MIPS-160 and IMPase 1 was assayed *in vitro*. Both purified enzymes are enzymatically active and their activity is highest at alkaline pH, showing a peak at pH 8.8 (Fig 5A). Hypertonic stress increases intracellular pH by activation of NHE exchangers to counteract shrinkage and facilitate regulatory volume increase (RVI) of vertebrate cells, for instance trout hepatocytes [44] and rat lymphocytes [45]. The normal range of intracellular pH in fish gill cells is between 7.5 and 8.5 and depends on the activity of NHE exchangers [46–48]. Because both tilapia enzymes are more active at alkaline pH and to stay within the normal range of intracellular pH, a pH of 8.2 was selected as the baseline for subsequent activity assays of both enzymes.

The catalytic specificity of both enzymes was validated using specific cofactors and inhibitors. As expected MIPS-160 activity depends on NAD^+^ (Fig 5B), and is greatly inhibited by 100 μM 2dG6P (Fig 5C). Likewise, IMPase 1 activity requires Mg^2+^ (Fig 5B), and is inhibited by both 50 μM L690,330 and 5 mM LiCl (Fig 5C).

### Kinetic properties of recombinant MIPS-160 and IMPase 1 *in vitro*

We determined the kinetic properties of both recombinant proteins in the standard buffers (MAB for MIPS-160, IAB for IMPase 1). As shown in Table 1, the *K*_M_ of MIPS-160 for G6*P* was 0.139 mM, the *k*_*cat*_ 0.208 sec^-1^ and the *k*_*cat*_/*K*_M_ 1.5 sec^-1^·mM^-1^. For IMPase 1, the *K*_M_ values where comparable when using Ins-1*P* and Ins-3*P* as substrates. However, the *k*_*cat*_ values for the two substrates were significantly different, being 0.37 times lower for Ins-3*P* than for Ins-1*P*. In this context, it is important to keep in mind that Ins-3*P* (but not Ins-1*P*) represents the product of MIPS catalysis and therefore, the substrate of IMPA in the Ins biosynthetic pathway that utilizes G6*P*. Substrate-specificity of IMPase 1 catalytic efficiency is also reflected in *k*_*cat*_/*K*_M_ values, which are 0.22 fold lower for Ins-3*P* compared to Ins-1*P*.

**Table 1:** Kinetic properties *in vitro* of the enzymes involved in Ins biosynthesis in *O*. *mossambicus*.

Enzyme	MIPS-160	IMPase 1	
**Substrate**	Glu-6*P*	Ins-1*P*	Ins-3*P*
***K*_M_**	0.139 ± 0.028	0.538 ± 0.074	0.93 ± 0.35
V_max_ *	0.00521± 0.0006	0.0073± 0.0007	0.0027± 0.0006
*k_cat_* *	0.2085 ± 0.012	2.585 ± 0.112	0.962 ± 0.102
*k_cat_*/*K*_M_*	1.5 ± 0.48	4.8 ± 1.1	1.05 ± 0.2

Initial rates with different substrate concentrations were measured *In vitro*, and kinetic properties (*K*_M_, V_max_ and *k*_*cat*_) were determined by non-linear regression analysis using GraphPad 5 software. Values for three separate experiments are shown (units: *K*_M_, mM ± SE; V_max_, nmol sec^-1^ ± SE; *k*_*cat*_, sec^-1^ ± SE; *k*_*cat*_/*K*_M_, sec^-1^·mM^-1^ ± SE). Eight substrate concentrations were used for determination of kinetic properties.

Asterisks represent parameters with significant differences (t-test, *P* < 0.05) for IMPase 1 assayed with different substrates (Ins-1*P* vs Ins-3*P*).

### Direct ionic effects on MIPS-160 and IMPase 1 activity

**Fig 6:**
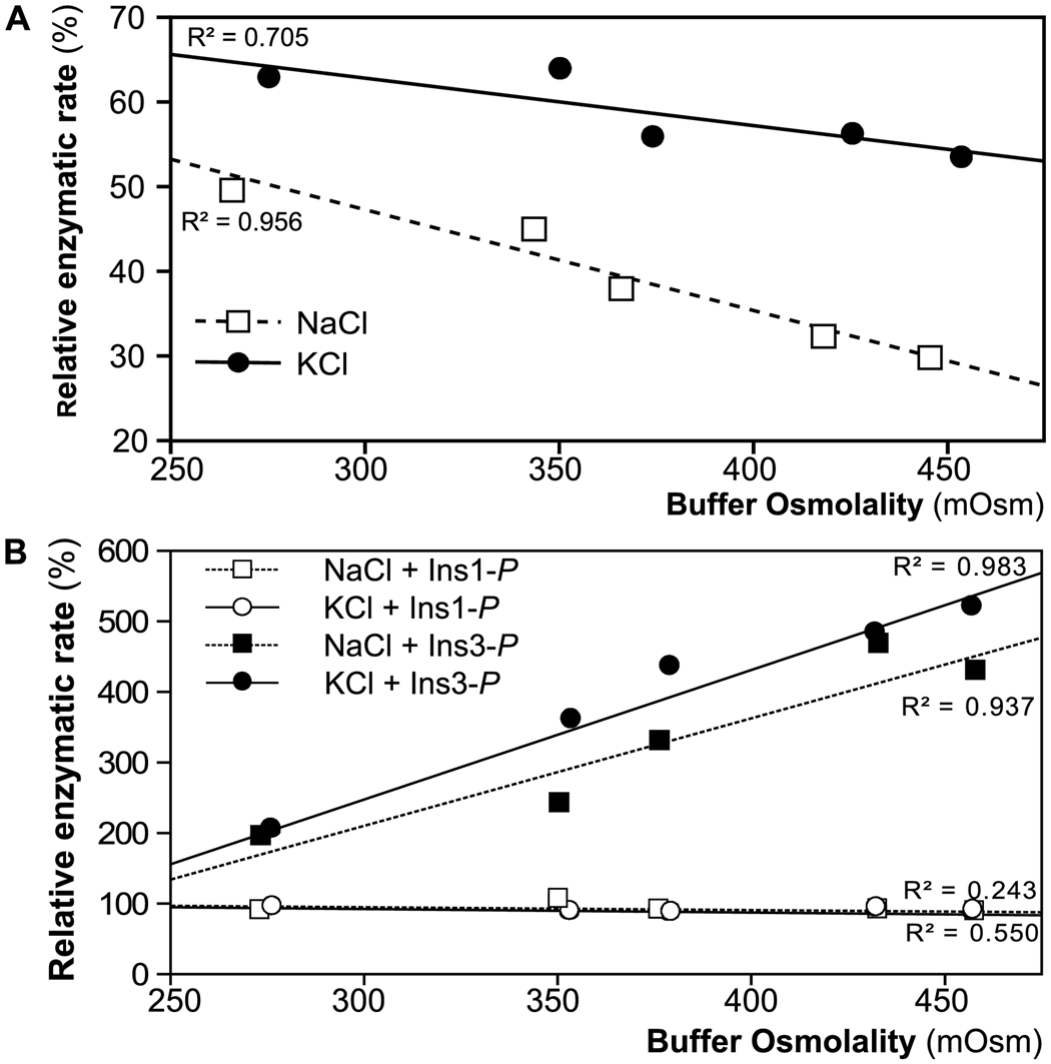
Enzymatic activity rate for MIPS-160 and IMPase 1 is modified under different osmotic conditions. MIPS-160 (**A**) and IMPase 1 (**B**) activity changes when concentration of ions (NaCl or KCl) is increased. Relative rate (activity compared to the activity in the assay buffer unsupplemented with ions) is graphed for the different osmolalities (achieved by addition of enough NaCl or KCl to reach the final osmolality). For IMPase 1, two different substrates (Ins-1 and Ins-3*P*) were assayed. In all cases, substrate concentration was 500 μM. Activity is expressed as relative expression (fold of change) compared to the activity in the unsupplemented SB.

To test for direct ionic effects on the catalytic efficiency of MIPS-160 and IMPase 1, we first determined the enzymatic activity rate of both MIPS-160 and IMPase 1 in buffers with different osmolalities. Osmolality variation relative to standard buffer conditions was achieved by varying the concentrations of either Na^+^ or K^+^ because these ions are most critical and relevant for cellular osmoregulation. Both MIPS-160 (Fig 6A) and IMPase 1 (Fig 6B) activities depend significantly on the osmotic and ionic (Na^+^ or K^+^) conditions. The relative activity rate of MIPS-160 decreases as osmolality increases, and this effect is more evident for NaCl compared to KCl, which means that it is very moderate *in vivo* where KCl concentrations are much higher than those of NaCl (Fig 6A). Inhibitory effects of increased NaCl (and to a lesser extent KCl) on enzyme activity are the rule and MIPS-160 is no exception in this regard [1]. However, our data show that IMPase 1 represents a very interesting exception to this general rule. When Ins-1*P* was used as the substrate IMPase 1 activity was unaffected by osmolality/ ionic strength over a wide range (Fig 6B). Even more surprising, when Ins-3*P* was used as the substrate, IMPase 1 activity increased significantly at higher osmolalities, independent of whether NaCl or KCl concentration was elevated (Fig 6B).

**Fig 7:**
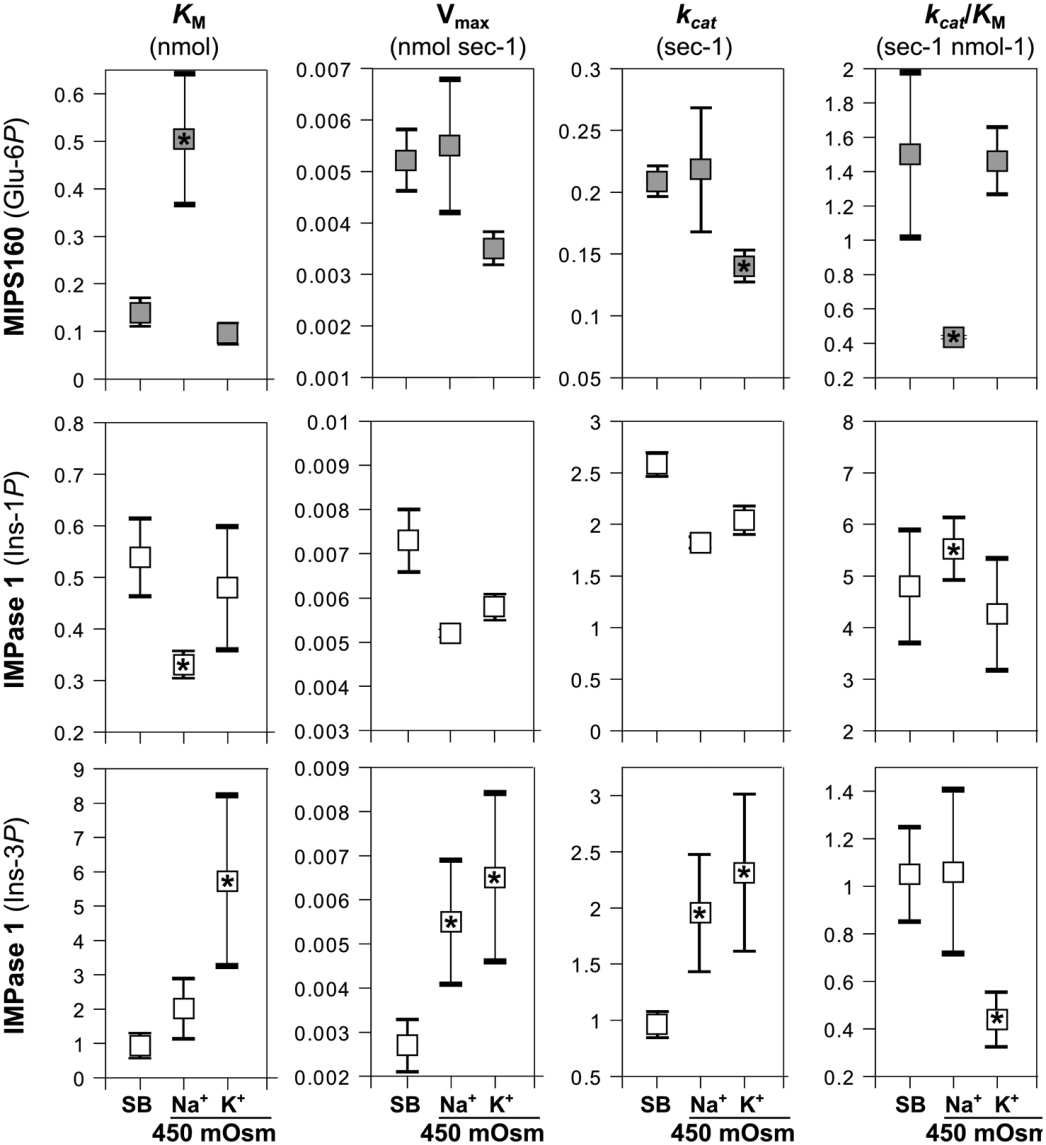
MIPS160 and IMPase 1 kinetics properties under different osmotic conditions. Kinetic properties were measured (in three separated experiments) under high osmolalities (450 mOsm), achieved by increase of either Na^+^ or K^+^ levels. The results are shown in absolute values and in fold of change compared to the values observed in the standard buffers (SB) with the basal osmolality (MAB = 124 mOsm; IAB = 220 mOsm). Five substrate concentrations were used to determine the kinetic properties (units: *K*_M_, mM ± SE; V_max_, nmol sec^-1^ ± SE; *k*_*cat*_, sec^-1^ ± SE; *k*_*cat*_/*K*_M_, sec^-1^·mM^-1^ ± SE). Asterisks denote significant differences with the values observed in MAB or IAB (*standard buffers*, SB) from three separate experiments (*t*-test, *P* < 0.05).

It has been shown previously that changes in ionic strength affect kinetic properties of enzymes [12]. Therefore, we determined the kinetic properties for both MIPS-160 and IMPase 1 at 450 mOsm hyperosmolality, which was achieved by addition of either Na^+^ or K^+^ as the cation and Cl^-^ as the anion (Fig 7). Compared to the properties observed in the standard buffer, MIPS-160 *K*_M_ was markedly increased when Na^+^ was added (3.85 fold higher) but not when K^+^ was added (Fig 7). In addition, *k*_*cat*_ was slightly reduced when K^+^ was added (0.68 fold), although the effect on the *k*_*cat*_/*K*_M_ ratio was negligible. These results suggest that the catalytic efficiency of MIPS-160 under hyperosmotic conditions is most susceptible to Na^+^ effects on K_M_. When using Ins-1*P* as the substrate of IMPase 1 and increasing osmolality by adding Na^+^ then *K*_M_ decreased 0.54 fold and the *k*_*cat*_/*K*_M_ ratio increased 1.78 fold but other kinetic properties (*k*_*cat*_, V_max_) were unaffected (Fig 7). Hyperosmolality by adding K^+^ had no effect on kinetic properties of IMPase 1 with Ins-1*P* as the substrate. When Ins-3*P* was used as the substrate and hyperosmolality was achieved by addition of NaCl then V_max_ and *k*_*cat*_ significantly increased (1.94 and 1.95 fold, respectively) (Fig 7). With Ins-3*P* as the substrate and hyperosmolality achieved by addition of KCl *K*_M_ and *k*_*cat*_ both increased significantly (6.27 and 2.3 fold, respectively), resulting in a net 60% decrease of the *k*_*cat*_/*K*_M_ ratio. These data illustrate that IMPase 1 kinetic properties are uniquely altered by hyperosmolality in a substrate- and ion-dependent manner.

## Discussion

In the present report, we cloned, expressed, purified and characterized the two enzymes that are required for Ins biosynthesis in Mozambique tilapia. Ins represents a compatible non nitrogenous osmolyte that protects macromolecules from hyperosmotic stress. Therefore, we have investigated direct ionic and osmotic effects on these two enzymes to gain insight into the mechanism by which Ins is accumulated in cells exposed to hyperosmolality.

### MIPS-160 and IMPase 1 sequence analysis

Several IMPase loci and at least two splice variants of MIPS have been identified in tilapia. To know which isoform/ variant of each gene represents the most suitable candidate for in-depth studies of osmotic and ionic effects on their activity, we used bioinformatics tools to reveal the most appropriate sequences. IMPase 1 mRNA and protein abundance increases much more strongly than that of other isoforms in different tissues of *O*. *mossambicus* [27, 28]. In addition, we have observed that the other two IMPase isoforms are expressed at very low levels and do not change in abundance during salinity stress (unpublished data). Likewise, the putative IMPA 1 orthologous On 1.1 showed the highest and most consistent induction of all four *IMPase* genes after exposure of *O*. *niloticus* to high salinity [29]. The closest orthologues of *O*. *mossambicus* IMPase 1 and *O*. *niloticus* On 1.1 are all sequences from fishes. Distinct from this group is another group of IMPases that are orthologous to human Hs 1.1. It is interesting that On 1.1 orthologues (including *O*. *mossambicus* IMPase 1) differ from other IMPase paralogues (Hs 1.1 sub-clade) by their reduced content of hydrophobic and increased content of negatively charged amino acids, which are both properties that have been shown previously to be characteristic of salt-tolerant proteins from extreme halophilic microorganisms [49, 50]. These properties stabilize protein 3D structures under high osmolality conditions while allowing retention of the flexibility required to proper interaction with substrates and cofactors [50, 51]. These data suggest that *O*. *mossambicus* IMPase 1 is an isoform that has been selected for production of Ins under high salinity conditions in fish.

For MIPS, one locus per genome is found, although many different isoforms (most likely resulting from alternative splicing of the mRNA) have been observed in several species. For example, in rat, at least eight MIPS variants have been detected either at the mRNA or protein level [23]. In humans, at least four *MIPS* variants have been observed at mRNA level [43]. In tilapia, two *MIPS* variants have been detected, MIPS-160 and MIPS-250. The latter harbors an addition of 29 amino acids, likely as a result of intron-retention during mRNA maturation [27, 28]. Whether these tilapia variants have different functions is currently unknown. In rats, however, the 16 kDa MIPS-γ variant acts as an inhibitor of the full length α isoform. Since the γ isoform only contains the NAD^+^ binding domain, the proposed mechanism involves competition for the cofactor between the two isoforms, resulting in a decreased activity of the full length isoform. The tilapia short variant (MIPS-160) was selected for cloning and characterization, for two main reasons. First, the extra 29 amino acid insertion in the MIPS-250 variant is not shared by any other of the isoforms analyzed (Fig 2), suggesting that this insert is not essential for function. Second, even though both variants were induced in tilapia tissues after salinity exposure, the MIPS-160 variant is expressed at higher levels and up-regulated to a greater extent [27].

The 3D models generated for both MIPS-160 and IMPase 1 (Fig 3A, S1 Table in Supporting Information) illustrate that the degree of sequence conservation is highest for amino acids surrounding the active site, while amino acids located at the protein periphery (solvent exposed) exhibit a lesser degree of conservation (Fig 3B). Based on these data we conclude that the functional constraints for these positions are highest with regard to their catalytic activity and not as high regarding the formation of oligomers or interaction with other proteins.

### Purified recombinant MIPS-160 and IMPase 1 are enzymatically active *in vitro*

Both proteins, expressed in *E*. *coli* and purified by alternative methods (Fig 4), are enzymatically active under the various conditions tested, and their activity required the presence of known cofactors: NAD^+^ for MIPS-160 and Mg^2+^ for IMPase 1 (Fig 5).

MIPS uses NAD^+^ as an additional active-site catalytic residue [52], participating in oxidation and reduction of intermediates during the catalytic process. Therefore, a cycle of NAD^+^→NADH→NAD^+^ occurs during catalysis, rendering no net production of NADH. However, NADH acts as a competitive inhibitor of MIPS, [53], since it can bind to the active site and interfere with the initial NAD^+^-mediated oxidation [54]. For IMPase, three atoms of a divalent cation are associated with the active site. The preferential divalent cation is Mg^2+^ [21, 55] although Mn^2+^ [56, 57] or Ca^2+^ [58] can substitute for Mg^2+^. Mg^2+^, which can bind with different affinities to each binding site in IMPase, is required for efficient substrate binding [59].

Our experiments utilizing known inhibitors of MIPS and IMPase prove that they are effective in inhibiting the tilapia enzymes. For MIPS, analogues to Glu-6*P* have been described as inhibitors, such as 2dG6*P* or glucitol-6*P* [60], both with a *K*_i_ in the μM concentration range [53, 54]. The substrate analogue 2-deoxy-glucitol-6P can bind to the active site of yeast MIPS and induce changes in the three-dimensional arrangement of the amino acids comprising the active site, suggesting that MIPS can attain an induced fit conformation during activation [61]. Valproate (VPA), a drug used in the therapeutic treatment of bipolar disorder was believed to inhibit MIPS in human brain at mM concentrations, since treatment with VPA caused a decrease in inositol levels in yeast [62] and mammalian brain [63]. However, in this study we did not observe any inhibitory effect of VPA on tilapia MIPS-160 (data not shown), which is consistent with studies on other species demonstrating that VPA does not inhibit MIPS directly [64]. It has recently been proposed that both yeast and human MIPS activity is regulated by phosphorylation, and that MIPS phosphorylation is influenced by VPA *in vivo* [65].

### MIPS-160 and IMPase 1 activity is directly regulated by the osmotic and ionic milieu

Enzymatic activity (and kinetic properties) are directly dependent in the physico-chemical milieu the enzymes are bathed in (i.e. pH, ionic milieu, temperature, etc.). In the particular case of ionic strength, it has been observed that the kinetic properties (such as *K*_M_ or V_max_) of various cytosolic enzymes are altered in the presence of inorganic solutes (such as NaCl or KCl), while similar concentration of compatible osmolytes (amino acids, inositol and even urea) had mild or no effects on enzymatic activity [1]. Considering that both MIPS-160 and IMPase 1 are enzymes involved in adaptation to hyperosmotic stress in fish, we decided to test their kinetic properties under different ionic and osmotic conditions. As a first step, we measured *K*_M_, V_max_ and *k*_*cat*_ for both enzymes in the standard IAB and MAB buffers (Table 1). Alteration of the osmotic and ionic milieu had similar effects on MIPS-160 as has been reported previously for other enzymes from different sources. However, notably, tilapia MIPS-160 activity was increased at high pH that is more characteristic of hypertonic conditions than normal cell conditions. For tilapia IMPase 1, we tested the effects of two different substrates (Ins-1*P* and Ins-3*P*) on enzyme activity. Interestingly, both V_max_ and the turnover constant *k*_*cat*_ (but not *K*_M_) were substrate-specific. These values were higher for Ins-1*P* than Ins-3*P*, suggesting a higher catalytic efficiency for Ins-1*P*, which is reflected in the higher *K*_M_/*k*_*cat*_ ratio for this substrate.

An important function for Ins is that it is a required precursor for Ins-containing phospholipid (phosphoinositide) biosynthesis [66]. In response to several stimuli, the Ins moiety of phosphoinositides (phosphorylated at several positions) is released and it acts as an intracellular second messenger. Moreover, different phosphorylation/dephosphorylation combinations may occur, giving rise to a number of differentially mono- or poly-phosphorylated versions of Ins, each with different functions [67]. For example, while Ins-3*P* is the product of MIPS activity, it can also be produced by dephosphorylation of different biphosphorylated Ins-1,3*P*_*2*_ or Ins-3,4*P*_*2*_, mediated by specific phosphatases [68]. However, the concentrations of these substrates are generally much lower than G6*P* and it is, therefore, unlikely that they will contribute significantly to the rise in Ins levels during hyperosmotic stress. Ins-1*P* can also be produced by alternative pathways, through dephosphorylation of inositol biphosphate precursors (such as Ins-1,3*P*_*2*_) or from hydrolysis of inositol (1:2) cyclic phosphate [69]. Previous reports for IMPase kinetic properties showed differential preferences for different substrates: IMPase purified from bovine brain showed *K*_M_ and V_max_ values for Ins-3*P* > Ins-1*P* [70], while for rat brain IMPase, *K*_M_ values differed in the order from Ins-5*P* > Ins-1*P* > Ins-4*P* and V_max_ from Ins-5*P* > Ins-4*P* > Ins-1*P* [71].

Measurement of the kinetic properties of IMPase 1 at increasing osmolality revealed two interesting facts: (1) the rate of enzymatic dephosphorylation increased for Ins-3*P* but not for Ins-1*P* when osmolality was elevated by increasing either K^+^ or Na^+^, and (2) the kinetic properties of IMPase 1 were altered when salinity was elevated and Ins-3*P* was used as the substrate (Fig 7). Specifically, *k*_*cat*_ (and consequently V_max_) were increased. We observed an increase of the *K*_M_ when KCl was used to elevate osmolality, as well as a similar (although insignificant) trend when NaCl was used to elevate osmolality to 450 mosmol/ kg. Thus, we conclude that the increase of *k*_*cat*_ explains the higher rates of IMPase activity observed for Ins-3*P* when medium osmolality is elevated by addition of either NaCl or KCl. When Ins-1*P* was used as the substrate, however, the *k*_*cat*_ was not affected, although we observed a decrease in the *K*_M_ when osmolality was elevated. Structural differences in how these substrates are bound to the active site may explain their different behavior with regard to IMPase 1 kinetic properties. When Ins-3*P* is bound to the active site, the orientation of its position 6 hydroxyl group favors the transition state (ES) and renders catalysis more efficient [21]. Interestingly, it has been proposed that aldose reductase is activated during hyperosmotic stress through an increase in V_max_ [10]. Aldose reductase catalyzes the conversion of D-glucose to the compatible osmolyte sorbitol in mammalian renal cells exposed to hypertonicity.

Tilapia IMPase 1 belongs to a clade of IMPases that can be distinguished from other vertebrate IMPase clades by properties (more negatively charged amino acids and a lower hydrophobic index) that resemble those of halophilic microorganisms. Proteins from such halophiles are more likely to be more rigid (in a “frozen” structure) at low salt concentration, but they gain flexibility and become more active at the proper osmotic and ionic strengths [51]. Based on this knowledge and the kinetic behavior discussed above, we suggest that tilapia IMPase 1 structural flexibility increases when binding Ins-3*P* (but not Ins-1*P*) at high ionic strength (hyperosmolality), allowing for more efficient catalysis.

Although MIPS-160 was not activated by hyperosmolality in the same way as IMPase 1 the inhibition of its activity was relatively small. Inhibition of MIPS activity by increasing concentrations of both K^+^ [72] and Na^+^ [73] is consistent with previous studies. Therefore, our data suggest that IMPase 1 is more rate-limiting for *Ins* biosynthesis under hyperosmotic conditions than MIPS-160. Nevertheless, both enzymes are activated under alkaline conditions (increased pH) and decreased H^+^ concentration. Increased ionic strength (Na^+^, K^+^) and increased pH are characteristic of intracellular conditions that result from hypertonicity and are retained even after RVI, which results from the activation of NHE and other transport proteins in response to hyperosmotic stress [10, 11]. In addition, *Ins* accumulation is further aided by glucose mobilization during salinity stress in tilapia [31, 74], which leads to increases in G6P, the immediate substrate of MIPS, which (together with activation by increased pH) could serve to offset the ionic inhibition of the catalytic efficiency of MIPS-160. Such conditions will strongly activate IMPase 1 and may also activate MIPS-160. The overall result of these direct ionic effects on IMPase 1 and MIPS-160 is a direct ionic stimulation of *Ins* biosynthesis.

In this study we have analyzed the effects of two different cations (Na^+^ and K^+^) and osmolality on MIPS and IMPase enzyme activity. We used Cl^-^ as the counter-ion and we should note that the intracellular concentration of this anion is somewhat lower than the ones employed in our experiments. Other ions, in particular divalent inorganic anions and organic anions contribute significantly to the overall anion concentration of live cells. Nevertheless, Cl^-^ is the most abundant anion in fish gill cells and it increases during hyperosmotic stress. For example, in fresh water acclimated *Salmo trutta*, Cl^-^ concentration is 51 mM in gill epithelial pavement cells, and 40 mM in mitochondria-rich cells [75]. In this species the intracellular Cl^-^ concentration increases to ca.80 mM in mitochondria-rich cells when fish are exposed to a salinity increase [75]. In Atlantic salmon, intracellular Cl^-^ concentration in gill cells is also high (80–120 mM) when they are exposed to hyperosmotic stress [76]. Even though the Cl^-^ counter-ion concentration used in our study is somewhat higher (approximately 175–225 mM) we observed strong cation-specific (Na^+^ versus K^+^) effects on IMPase activity suggesting that the effect of the counter ion is minimal. It is also noteworthy that the osmolality range used in our experiments falls in line with previously reported physiological plasma osmolality data for tilapia [28]. It is clear that the ionic composition of biological tissues is far more complex than the buffered solution used in our study. Therefore, it might be interesting to study effects of other ions on the activity of these two enzymes in the future.

In summary, based on the results of this study and the evidence discussed above we propose that direct ionic regulation of *Ins* biosynthesis enzymes in tilapia provides a mechanism for efficient and rapid accumulation of the compatible organic osmolyte *Ins* during hyperosmotic stress. As the concentration of *Ins* increases during its accumulation it gradually replaces excessive inorganic ions by filling the osmotic gap and restoring intracellular pH to eventually attain a normal intracellular milieu. Thus, *Ins* accumulation itself removes the initial stress (increased inorganic ion strength) that stimulates its biosynthesis. This system represents a straight-forward and efficient biochemical feedback loop that is driven by ionic effects on *Ins* biosynthesis enzymes for directly controlling the intracellular concentration of compatible organic osmolytes.

## Supporting Information

S1 Fig MSA (T-COFFEE) and phylogenetic tree (PhyML; http://www.phylogeny.fr/version2_cgi/one_task.cgi?task_type=phyml) used for ConSurf analysis of IMPase sequences. Colors in MSA represent the conservation assigned per column.
(PDF)

S2 Fig MSA (T-COFFEE) and phylogenetic tree (PhyML; http://www.phylogeny.fr/version2_cgi/one_task.cgi?task_type=phyml) used for ConSurf analysis of MIPS sequences. Colors in MSA represent the conservation assigned per column.
(PDF)

S1 Table Quality parameters of MIPS-160 and IMPase 1 3D structural models resulting from I-TASSER prediction. The best models (rank 1) are shown in Fig 3A.
(PDF)

S2 Table Bidirectional Best BLAST hit to determine putative IMPase orthologues. The sequence for *H*. *sapiens* (left) or *O*. *niloticus* (right) IMPase was used to look for the best hits in the several non-fish and fish species. The search was performed using NCBI BLAST and RefSeq databases, unless specified otherwhise (* = Search on Ensembl BLAST/BLAT; ** = Search vs non-redundant databases). The first hit for each species (Column A) was used to search in the database corresponding to the query used for the first search (i.e., against human for the left table, against Nile tilapia in the right column). If the first hit matched the identity of the sequence used in the original BLAST, the sequences were considered orthologues (in red, the non orthologues sequences).
(PDF)

S3 Table Annotations in databases and accession numbers of sequences used to build the IMPase’s phylogenetic tree in Fig 1. The annotations extracted from the databases are shown. The colored sequences correspond to the putative orthologues to human IMPase (red) and osmotic stress responsive tilapia’s IMPase (blue). In addition, the annotations of some of the sequences employed in Kalujnaia et al (2013) are shown for *O*. *niloticus* and *A*. *anguilla* annotations
(PDF)

S4 Table MIPS sequences and annotations used to build the multiple sequence analysis shown in Fig 2. The sequence of OmMIPS250 is presented (highlighted region corresponding to the additional fragment in comparison to the MIPS160 version).
(PDF)

S5 Table Primers used for cloning MIPS-160 and IMPase 1 from Mozambique tilapia's gills. The sequence of the restriction enzyme sites (RES) included for cloning are underlined.
(PDF)
