# Supplementary material for: Direct Ionic Regulation of the Activity of Myo-Inositol Biosynthesis Enzymes in Mozambique Tilapia
Source: PLoS One. 2015 Jun 11;10(6):e0123212. doi: 10.1371/journal.pone.0123212 (PMC4466255; doi:10.1371/journal.pone.0123212)
Supplement: S1 Fig — Colors in MSA represent the conservation assigned per column. (PDF) [file pone.0123212.s001.pdf]

SUPPLEMENTARY MATERIAL

Direct ionic regulation of the activity of *myo*-inositol biosynthesis enzymes in Mozambique tilapia

S1 Figure

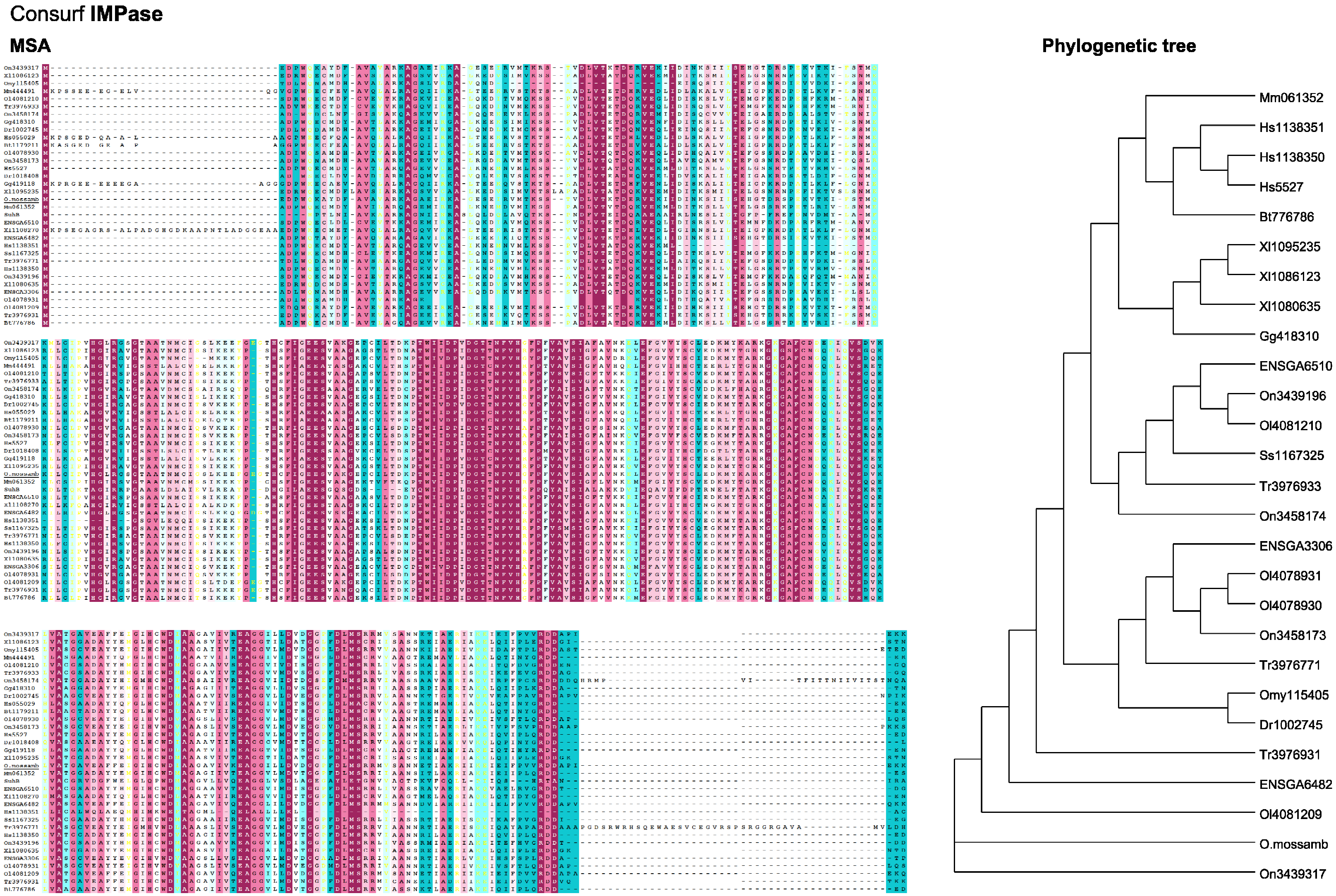

S1 Figure. MSA (T-COFFEE) and phylogenetic tree (PhyML; http://www.phylogeny.fr/version2_cgi/one_task.cgi?task_type=phyml) used for ConSurf analysis of IMPase sequences. Colors in MSA represent the conservation assigned per column.
