# Supplementary material for: Direct Ionic Regulation of the Activity of Myo-Inositol Biosynthesis Enzymes in Mozambique Tilapia
Source: PLoS One. 2015 Jun 11;10(6):e0123212. doi: 10.1371/journal.pone.0123212 (PMC4466255; doi:10.1371/journal.pone.0123212)
Supplement: S1 Table — The best models (rank 1) are shown in Fig 3A. (PDF) [file pone.0123212.s003.pdf]

SUPPLEMENTARY MATERIAL

Direct ionic regulation of the activity of *myo*-inositol biosynthesis enzymes in Mozambique tilapia

S1 Table

MIPS-160							IMPase 1						
**Rank**	**PDB Hit**	**C-score[a]**	**TM-score[b]**	**RMSD[c]**	**IDEN[d]**	**Cov.[e]**	**Rank**	**PDB Hit**	**C-score[a]**	**TM-score[b]**	**RMSD[c]**	**IDEN[d]**	**Cov.[e]**
1	1vkoA	0.39	0.9	1.36	0.535	0.913	1	2hhmA	1.37	0.957	0.42	0.625	0.961
2	1p1jA	-0.75	0.881	1.81	0.538	0.908	2	2bjiA	-3.39	0.955	0.75	0.62	0.968
3	1jkfB	-0.15	0.77	1.99	0.516	0.801	3	2cziA	-5	0.876	1.34	0.461	0.905

(a) Confidence score for estimating the quality of predicted models. C-score is typically in the range of [-5,2], the higher the C-score, the higher the confidence of the model.

(b)TM-score is a measure of global structural similarity between query and template protein (from 0 to 1).

(c) Root Mean Square Deviation between residues that are structurally aligned by TM-align.

(d) IDEN is the percentage sequence identity in the structurally aligned region.

(e) Coverage of the alignment by TM-align and is equal to the number of structurally aligned residues divided by length of the query protein.

S1 Table. Quality parameters of MIPS-160 and IMPase 1 3D structural models resulting from I-TASSER prediction. The best models (rank 1) are shown in figure 3A.
