# Supplementary material for: Direct Ionic Regulation of the Activity of Myo-Inositol Biosynthesis Enzymes in Mozambique Tilapia
Source: PLoS One. 2015 Jun 11;10(6):e0123212. doi: 10.1371/journal.pone.0123212 (PMC4466255; doi:10.1371/journal.pone.0123212)
Supplement: S2 Table — The sequence for H. sapiens (left) or O. niloticus (right) IMPase was used to look for the best hits in the several non-fish and fish species. The search was performed using NCBI BLAST and RefSeq databases, unless specified otherwhise (* = Search on Ensembl BLAST/BLAT; ** = Search vs non-redundant databases). The first hit for each species (Column A) was used to search in the database corresponding to the query used for the first search (i.e., against human for the left table, against Nile tilapia in the right column). If the first hit matched the identity of the sequence used in the original BLAST, the sequences were considered orthologues (in red, the non orthologues sequences). (PDF) [file pone.0123212.s004.pdf]

SUPPLEMENTARY MATERIAL

Direct ionic regulation of the activity of *myo*-inositol biosynthesis enzymes in Mozambique tilapia

S2 Table

Query Hs 1.1			Query On 1.X1		
Using *Homo sapiens* NM_005536.3 vs	**blastx (vs refseq, ** vs NR)**	Using the 1st hit vs human refseq RNA (tblastn)	USING *O. niloticus* XP_003439317.1	**blastP (vs refseq, ** vs NR)**	Using the 1st hit vs *O. niloticus* refseq protein (blastp)
	1st hit on species Database	1st hit on *H. sapiens*		1st hit on species Database	1st hit on *O. niloticus*
Bos taurus	NP_776786.1	**NM_005536.3**	*Bos taurus*	NP_776786.1	XP_003439196.1
Mus musculus	NP_061352.2	**NM_005536.3**	*Mus musculus*	NP_061352.2	XP_003439196.1
Gallus gallus	XP_418310.2	**NM_005536.3**	*Gallus gallus*	XP_418310.3	XP_003439196.1
Homo sapiens	NP_001138350.1	**NM_005536.3**	*Homo sapiens*	NP_005527.1	XP_003439196.1
Xenopus laevis	NP_001080635.1	**NM_005536.3**	*Xenopus laevis*	NP_001080635.1	XP_003439196.1
Oryzias latipes	XP_004081210.1	**NM_005536.3**	*Salmo salar***	NP_001266101.1	XP_003458173.1
Salmo salar	NP_001167325.1	**NM_005536.3**	*Danio rerio*	NP_001002745.1	XP_003458173.1
Danio rerio	NP_001002745.1	**NM_005536.3**	*Oryzias latipes*	XP_004081209.1	**XP_003439317.1**
Oreochromis niloticus	XP_003439196.1	**NM_005536.3**	*Oreochromis mossambicus*	AFY10067.1	**XP_003439317.1**
Takifugu rubripes	XP_003976933.1	**NM_005536.3**	*Takifugu rubripes*	XP_003976931.1	**XP_003439317.1**
Xiphophorus maculatus	XP_005805762.1	**NM_005536.3**	*Xiphophorus maculatus*	XP_005816948.1	**XP_003439317.1**
*Gasterosteus aculeatus**	ENSGACP00000006493	**NM_005536.3**	*Anguilla anguilla***	CBI68709.1	**XP_003439317.1**
*Anguilla anguilla***	CCP46952.1	**NM_005536.3**	*Gasterosteus aculeatus**	ENSGACP00000006465	**XP_003439317.1**
*Tetraodon nigroviridis**	ENSTNIG00000013660	**NM_005536.3**	*Tetraodon nigroviridis**	CAG05496.1	**XP_003439317.1**

S2 Table. Bidirectional Best BLAST hit to determine putative IMPase orthologues. The sequence for *H. sapiens* (left) or *O. niloticus* (right) IMPase was used to look for the best hits in the several non-fish and fish species. The search was performed using NCBI BLAST and RefSeq databases, unless specified otherwise (* = Search on Ensembl BLAST/BLAT; ** = Search vs non-redundant databases). The first hit for each species (Column A) was used to search in the database corresponding to the query used for the first search (i.e., against human for the left table, against Nile tilapia in the right column). If the first hit matched the identity of the sequence used in the original BLAST, the sequences were considered orthologues (in red, the non orthologues sequences).
