# Supplementary material for: Direct Ionic Regulation of the Activity of Myo-Inositol Biosynthesis Enzymes in Mozambique Tilapia
Source: PLoS One. 2015 Jun 11;10(6):e0123212. doi: 10.1371/journal.pone.0123212 (PMC4466255; doi:10.1371/journal.pone.0123212)
Supplement: S3 Table — The annotations extracted from the databases are shown. The colored sequences correspond to the putative orthologues to human IMPase (red) and osmotic stress responsive tilapia’s IMPase (blue). In addition, the annotations of some of the sequences employed in Kalujnaia et al (2013) are shown for O. niloticus and A. anguilla annotations (PDF) [file pone.0123212.s005.pdf]

SUPPLEMENTARY MATERIAL

Direct ionic regulation of the activity of *myo*-inositol biosynthesis enzymes in Mozambique tilapia

S3 Table

Species	*Description (annotation at NCBI)*	*Accession*	*Name in tree*	*Label in Kalujnaia et al. (2013)*
Bos taurus	inositol monophosphatase 1 [Bos taurus] >ref|XP_005215709.1| PREDICTED: inositol monophosphatase 1 isoform X2 [Bos taurus] >ref|XP_005215710.1| PREDICTED: inositol monophosphatase 1 isoform X3 [Bos taurus]	NP_776786.1	Bt_1.2	
	PREDICTED: inositol monophosphatase 1 isoform X1 [Bos taurus]	XP_005215708.1	Bt_1.1	
	inositol monophosphatase 2 [Bos taurus]	NP_001179211.1	Bt_2	
Homo sapiens	**inositol monophosphatase 1 isoform 1 [Homo sapiens]**	**NP_005527.1**	Hs_1.1	
	**inositol monophosphatase 1 isoform 2 [Homo sapiens]**	**NP_001138350.1**	Hs_1.2	
	**inositol monophosphatase 2 [Homo sapiens]**	**NP_055029.1**	Hs_2	
	**inositol monophosphatase 1 isoform 3 [Homo sapiens]**	**NP_001138351.1**	Hs1.3	
Gallus gallus	PREDICTED: inositol monophosphatase 1 isoform X5 [Gallus gallus]	XP_004939993.1	Gg_1.5	
	PREDICTED: inositol monophosphatase 1 isoform X3 [Gallus gallus]	XP_418310.3	Gg_1.3	
	PREDICTED: inositol monophosphatase 1 isoform X4 [Gallus gallus]	XP_004939992.1	Gg_1.4	
	PREDICTED: inositol monophosphatase 2 [Gallus gallus]	XP_419118.1	Gg_2	
Xenopus laevis	**inositol(myo)-1(or 4)-monophosphatase 1 [Xenopus laevis]**	**NP_001080635.1**	Xl_1	
	**inositol(myo)-1(or 4)-monophosphatase 1 [Xenopus laevis]**	**NP_001086123.1**	Xl_1.1	
	**inositol monophosphatase 1 [Xenopus laevis]**	**NP_001095235.1**	Xl_1.2	
	**inositol(myo)-1(or 4)-monophosphatase 2 [Xenopus laevis]**	**NP_001108270.1**	Xl_2	
Oryzias latipes	PREDICTED: inositol monophosphatase 1-like [Oryzias latipes]	XP_004081209.1	Ol_1	
	PREDICTED: inositol monophosphatase 1-like isoform 1 [Oryzias latipes]	XP_004078930.1	Ol_1.1	
	PREDICTED: inositol monophosphatase 1-like isoform 2 [Oryzias latipes]	XP_004078931.1	Ol_1.3	
	PREDICTED: inositol monophosphatase 1-like [Oryzias latipes]	XP_004081210.1	Ol_1.4	
Takifugu rubripes	**PREDICTED: inositol monophosphatase 1-like [Takifugu rubripes]**	**XP_003976931.1**	Tr_1	
	**PREDICTED: inositol monophosphatase 1-like [Takifugu rubripes]**	**XP_003976771.1**	Tr_1.1	
	**PREDICTED: inositol monophosphatase 1-like [Takifugu rubripes]**	**XP_003976933.1**	Tr_1.3	
Danio rerio	inositol monophosphatase 1 [Danio rerio] >ref|XP_005163453.1| PREDICTED: inositol monophosphatase 1 isoform X1 [Danio rerio] >ref|XP_005163454.1| PREDICTED: inositol monophosphatase 1 isoform X2 [Danio rerio] >ref|XP_005163455.1| PREDICTED: inositol monophosphatase 1 isoform X3 [Danio rerio]	NP_001002745.1	Dr_1	

	inositol monophosphatase 2 [Danio rerio]	NP_001018408.1	Dr_2	
Oreochromis niloticus	**PREDICTED: inositol monophosphatase 1-like isoform X1 [Oreochromis niloticus] >ref|XP_005449080.1| PREDICTED: inositol monophosphatase 1-like isoform X2 [Oreochromis niloticus] >ref|XP_005449081.1| PREDICTED: inositol monophosphatase 1-like isoform X3 [Oreochromis niloticus]**	**XP_003439317.1**	On_1.1	IMPA 1.1
	**PREDICTED: inositol monophosphatase 1-like isoform X1 [Oreochromis niloticus] >ref|XP_005476952.1| PREDICTED: inositol monophosphatase 1-like isoform X2 [Oreochromis niloticus]**	**XP_003458173.1**	On_1.3	IMPA 1.3
	**PREDICTED: inositol monophosphatase 1-like [Oreochromis niloticus]**	**XP_003439196.1**	On_1.2	IMPA 1.2
	**PREDICTED: inositol monophosphatase 1-like isoform X2 [Oreochromis niloticus] >ref|XP_005476948.1| PREDICTED: inositol monophosphatase 1-like isoform X3 [Oreochromis niloticus]**	**XP_005476947.1**	On_1.4	IMPA 1.4
Anguilla anguilla	inositol(myo)-1(or 4)-monophosphatase 1 [Anguilla anguilla]	CBI68709.1	Aa_1.1	IMPA 1.1
	inositol monophosphatase [Anguilla anguilla]	CCP46953.1	Aa_1.3	IMPA 1.3
	inositol monophosphatase [Anguilla anguilla]	CCP46952.1	Aa_1.2	IMPA 1.2
	inositol monophosphatase [Anguilla anguilla]	CCP46954.1	Aa_2	IMPA 2
Salmo salar	**inositol monophosphatase [Salmo salar] >emb|CCP46955.1| inositol monophosphatase [Salmo salar]**	**NP_001266101.1**	Ss_1	
	**inositol monophosphatase 1 [Salmo salar] >gb|ACN11363.1| Inositol monophosphatase [Salmo salar]**	**NP_001167325.1**	Ss_1.1	
Tetraodon nigroviridis	unnamed protein product [Tetraodon nigroviridis]	CAG05496.1	Tn_1.1	
	unnamed protein product [Tetraodon nigroviridis]	CAF90281.1	Tn_1.2	
	unnamed protein product [Tetraodon nigroviridis]	CAG05495.1	Tn_1.3	
	inositol(myo)-1(or 4)-monophosphatase 1 [Source:HGNC Symbol;Acc:HGNC:6050]	ENSTNIG00000013660	Tn_1.4	
X. maculatus	**PREDICTED: inositol monophosphatase 1-like [Xiphophorus maculatus]**	**XP_005816948.1**	Xm_1.1	
	**PREDICTED: inositol monophosphatase 1-like [Xiphophorus maculatus]**	**XP_005805762.1**	Xm_1.2	
Gasterosteus aculeatus	inositol(myo)-1(or 4)-monophosphatase 1 [Source:HGNC Symbol;Acc:6050]	ENSGACP00000006465	Ga_1	
	inositol(myo)-1(or 4)-monophosphatase 1 [Source:ZFIN;Acc:ZDB-GENE-040718-245]	ENSGACP00000003295	Ga_1.1	
	inositol(myo)-1(or 4)-monophosphatase 1 [Source:HGNC Symbol;Acc:6050]	ENSGACP00000006493	Ga_1.2	
**Outgroup**	suhB myo-inositol-1(or 4)-monophosphatase [Azoarcus sp. KH32C]	YP_007552102.1	suhB	

S3 Table. Annotations in databases and accession numbers of sequences used to build the IMPase's phylogenetic tree in figure 1. The annotations extracted from the databases are shown. The colored sequences correspond to the putative orthologues to human IMPase (red) and osmotic stress responsive tilapia's IMPase (blue). In addition, the annotations of some of the sequences employed in Kalujnaia et al (2013) are shown for *O. niloticus* and *A. anguilla* annotations .
