# Supplementary material for: Direct Ionic Regulation of the Activity of Myo-Inositol Biosynthesis Enzymes in Mozambique Tilapia
Source: PLoS One. 2015 Jun 11;10(6):e0123212. doi: 10.1371/journal.pone.0123212 (PMC4466255; doi:10.1371/journal.pone.0123212)
Supplement: S4 Table — The sequence of OmMIPS250 is presented (highlighted region corresponding to the additional fragment in comparison to the MIPS160 version). (PDF) [file pone.0123212.s006.pdf]

SUPPLEMENTARY MATERIAL

Direct ionic regulation of the activity of *myo*-inositol biosynthesis enzymes in Mozambique tilapia

S4 Table

Species	*Label in MSA*	*Accession number*	*Length*	*Description*	*Notes*
Oreochromis mossambicus	OmMIPS160	ABE98241.1	518	Oreochromis mossambicus clone SSH #160 myo-inositol-1 phosphate synthase-like	OmMIPS variant 160
	OmMIPS250	**	547	NA	OmMIPS variant 250
Homo sapiens	HsMIPS_1	NP_057452.1	558	inositol-3-phosphate synthase 1 isoform 1 [Homo sapiens]	Human variant 1
	HsMIPS_2	NP_001164409.1	504	inositol-3-phosphate synthase 1 isoform 2 [Homo sapiens]	Human variant 2
	HsMIPS_4	NM_001253389.1	430	inositol-3-phosphate synthase 1 isoform 4 [Homo sapiens]	Human variant 4
Takifugu rubripes	Fugu	XP_003962824.1	550	PREDICTED: inositol-3-phosphate synthase 1-A-like [Takifugu rubripes]	
Xenopus laevis	Xenopus	NP_001086783.2	563	inositol-3-phosphate synthase 1-B [Xenopus laevis]	
Salmo salar	Salmon	NP_001133802	551	Inositol-3-phosphate synthase A [Salmo salar]	
Mus musculus	Mouse	NP_076116	557	inositol-3-phosphate synthase 1 [Mus musculus]	
Rattus norvegicus	Rat α	NP_001013902.2	557	inositol-3-phosphate synthase 1 [Rattus norvegicus]	Rat α isoform
	Rat β	***	539	inositol-3-phosphate synthase 1 [Rattus norvegicus]	Rat β isoform
	Rat γ		148	inositol-3-phosphate synthase 1 [Rattus norvegicus]	Rat γ isoform
Saccharomyces cerevisiae	Yeast	NP_012382.2	533	inositol-3-phosphate synthase INO1 [Saccharomyces cerevisiae S288c]	

** sequence derived from cloning and sequencing

```
>OmMIPS 250
MSVNVHINSPNVKYTDSHIEAQYSYQTTSVHRDGNKVTVTPRTTEMTIRTERRVTRLGVMLVGWGGNNGTTVTAAVLANKMGLTWKTKNGVKKANYFGSLLQSSTVCLGSGLE
GEVNVPFRDLLPMVHPNDIVFDGWDISSLDLGSAMERAQVLDWSLQEQLRPYMSCLKPRPSIYIPEFIAANQESRADNVLTGTMAEQVIKVSDSPFYSSVYFFSLAICLKECM
IFQMERIRADIRDFRQASGVDKVIVLWTANTERFCDIIPGVNDSAKNLLAAIQAGAEASPSTLFVVASILEGCAYINGSPQNTFVPGAIELAMQRGVFIGGDDFKSGQTKIKS
VLVDFLVSAGIKPTSIVSYNHLGNNDGKNLSAPQQFRSKEISKSNVVDDMVQSNPILYEPGEKPDHCVVIKYVPYVGDSKRAMDEYTSEIMMGGINTIALHNTCEDSLLATPI
ILDLVMLTELCQRVTIKPQGEESFQSFHSVLSLLSFLCKAPLVPSGTPVVNAFFRQRASIENIMRACLGLPPQNHMLLEHKLQRNFLPPHETCVNNDVASLKKVPLVNGNHIP
LTNGVYAHMDHTACAL
```

*** derived from Seelan, R. S., Lakshmanan, J., Casanova, M. F. and Parthasarathy, R. N. (2009) Identification of myo-Inositol-3-phosphate Synthase Isoforms: CHARACTERIZATION, EXPRESSION, AND PUTATIVE ROLE OF A 16-kDa γc ISOFORM. Journal of Biological Chemistry. 284, 9443-9457

S4 Table. MIPS sequences and annotations used to build the multiple sequence analysis shown in figure 2. The sequence of OmMIPS250 is presented (highlighted region corresponding to the additional fragment in comparison to the MIPS160 version).
