# Supplementary material for: Direct Ionic Regulation of the Activity of Myo-Inositol Biosynthesis Enzymes in Mozambique Tilapia
Source: PLoS One. 2015 Jun 11;10(6):e0123212. doi: 10.1371/journal.pone.0123212 (PMC4466255; doi:10.1371/journal.pone.0123212)
Supplement: S5 Table — The sequence of the restriction enzyme sites (RES) included for cloning are underlined. (PDF) [file pone.0123212.s007.pdf]

SUPPLEMENTARY MATERIAL
Direct ionic regulation of the activity of *myo*-inositol biosynthesis enzymes in Mozambique tilapia

S5 Table

Goal	Name	Sequence (5'-3')	Features
Cloning MIPS-160	OmMIPS_NheF	CGCTAGCTCCGTGAACGTTCACATCAAC	Binds in 5' end – Adds Nhe I RES
	OmMIPS_XhoR	CCTCGAGTAATGCACATGCTGTGTGGTC	Binds in 3' end – Adds Xho I RES – Excludes native Stop codon
Cloning IMPase 1	OmIMPase_NheF	CGCTAGCGAAGATCCATGGCAGAAGGC	Binds in 5' end – Adds Nhe I RES
	OmIMPase_XhoR	CCTCGAGCTTCTTCTCTATTGGAGCGTCATCC	Binds in 3' end – Adds Xho I RES – Excludes native Stop codon

S5 Table. Primers used for cloning MIPS-160 and IMPase 1 from Mozambique tilapia's gills. The sequence of the restriction enzyme sites (RES) included for cloning are underlined.
